# Effects of E-Learning in a Continuing Education Context on Nursing Care: Systematic Review of Systematic Qualitative, Quantitative, and Mixed-Studies Reviews

**DOI:** 10.2196/15118

**Published:** 2019-10-02

**Authors:** Geneviève Rouleau, Marie-Pierre Gagnon, José Côté, Julie Payne-Gagnon, Emilie Hudson, Carl-Ardy Dubois, Julien Bouix-Picasso

**Affiliations:** 1 Faculty of Nursing Université Laval Quebec, QC Canada; 2 University of Montreal Hospital Research Centre Montreal, QC Canada; 3 Centre de Recherche sur les Soins et les Services de Première Ligne de l'Université Laval Quebec, QC Canada; 4 Faculty of Nursing Université de Montréal Montreal, QC Canada; 5 School of Nursing McGill University Montreal, QC Canada; 6 Public Health Research Institute Université de Montréal Montreal, QC Canada; 7 Department of Management, Evaluation and Health Policy School of Public Health Université de Montréal Montreal, QC Canada; 8 Education and Health Practices Laboratory Paris 13 University Sorbonne Paris Cité University Paris France; 9 Department of Education for Non-Medical Personnel French Military Health Service Academy École du Val-de-Grâce Paris France

**Keywords:** continuing education, e-learning, nurses, nursing care, systematic review of systematic reviews

## Abstract

**Background:**

E-learning is rapidly growing as an alternative way of delivering education in nursing. Two contexts regarding the use of e-learning in nursing are discussed in the literature: (1) education among nursing students and (2) nurses’ continuing education within a life-long learning perspective. A systematic review of systematic reviews on e-learning for nursing and health professional students in an academic context has been published previously; however, no such review exists regarding e-learning for registered nurses in a continuing education context.

**Objective:**

We aimed to systematically summarize the qualitative and quantitative evidence regarding the effects of e-learning on nursing care among nurses in a continuing education context.

**Methods:**

We conducted a systematic review of systematic qualitative, quantitative, and mixed-studies reviews, searching within four bibliographic databases. The eligibility criteria were formulated using the population, interventions, comparisons, outcomes, and study design (PICOS) format. The included population was registered nurses. E-learning interventions were included and compared with face-to-face and any other e-learning interventions, as well as blended learning. The outcomes of interest were derived from two models: nursing-sensitive indicators from the Nursing Care Performance Framework (eg, teaching and collaboration) and the levels of evaluation from the Kirkpatrick model (ie, reaction, learning, behavior, and results).

**Results:**

We identified a total of 12,906 records. We retrieved 222 full-text papers for detailed evaluation, from which 22 systematic reviews published between 2008 and 2018 met the eligibility criteria. The effects of e-learning on nursing care were grouped under Kirkpatrick’s levels of evaluation: (1) nurse reactions to e-learning, (2) nurse learning, (3) behavior, and (4) results. Level 2, nurse learning, was divided into three subthemes: knowledge, skills, attitude and self-efficacy. Level 4, results, was divided into patient outcomes and costs. Most of the outcomes were reported in a positive way. For instance, nurses were satisfied with the use of e-learning and they improved their knowledge. The most common topics covered by the e-learning interventions were medication calculation, preparation, and administration.

**Conclusions:**

The effects of e-learning are mainly reported in terms of nurse reactions, knowledge, and skills (ie, the first two levels of the Kirkpatrick model). The effectiveness of e-learning interventions for nurses in a continuing education context remains unknown regarding how the learning can be transferred to change practice and affect patient outcomes. Further scientific, methodological, theoretical, and practice-based breakthroughs are needed in the fast-growing field of e-learning in nursing education, especially in a life-learning perspective.

**Trial Registration:**

International Prospective Register of Systematic Reviews (PROSPERO) CRD42016050714; https://www.crd.york.ac.uk/prospero/display_record.php?RecordID=50714

## Introduction

### Background

E-learning is rapidly growing as an alternative way of delivering education [1,2]. Nicoll et al [3] used the term *technology-enhanced learning* and stated that “it is a means by which learners can be provided with enhanced or transformed educational experiences.” Many other terms have been used synonymously and interchangeably to designate e-learning, such as computer-assisted learning, online learning, or Web-based learning [4]. For the purpose of this paper, we will use *e-learning* as an umbrella term to entail a variety of electronic, digital, or mobile devices used to support learning [5]. Clark and Mayer [6] specify elements about the *what*, *how*, and *why* of e-learning. The *what* includes content and instructional methods. The *how* encompasses elements such as the format (eg, asynchronous and webinars) and the use of multimedia (eg, video, animation, and printed words). The *why* is about, for instance, the achievement of learning objectives and/or the performance of skills applied in a workplace context.

In the literature targeting the use of e-learning in nursing, two populations and contexts are discussed. The first one is education among nursing students (eg, in Voutilainen et al [7]) who participate in educational programs mainly offered in academic settings. For instance, undergraduate nursing students have to develop entry-level competencies to meet the practice expectations required in getting their registered nurse (RN) licensure in order to “provide safe, competent, compassionate, and ethical nursing care in a variety of practice settings” [8]. The second context is continuing education (CE), also called continuing professional development [9] or continuing competency [8], targeting a life-long learning perspective and staff development (eg, Knapp and Byers [10]). RNs have to meet CE expectations to be eligible to renew their licensure and registration each year, with the goals of acquiring new competencies, maintaining the acquired ones, enhancing their practice, and keeping their skills relevant and up-to-date [8]. We refer here to CE programs that are applicable in workplace settings. The use of e-learning by nurses in a CE context is the one that retained our attention [11] for two main reasons: much more attention has been given to nursing students than to RNs [5,12] and this CE context will be informative to lay the groundwork for a wider research project.

A previous systematic review of systematic reviews (SRSRs) of e-learning for nursing and health professional students in an academic context has been conducted [5,12]. The findings show that e-learning is equivalent to traditional learning. However, e-learning has proven to have large effects compared to no intervention in health professions [13]. To the best of our knowledge, we found no SRSRs about e-learning used by RNs in a CE context.

### Objective

We aimed to systematically summarize the qualitative and quantitative evidence that comes from systematic qualitative, quantitative, and mixed-studies reviews regarding the effects of e-learning on nursing care among nurses in a CE context.

## Methods

The protocol was registered in the International Prospective Register of Systematic Reviews (PROSPERO) (number: CRD42016050714) and was published elsewhere [11].

### Design

We conducted a systematic review (SR) of systematic qualitative, quantitative, and mixed-studies reviews with the intent of bringing together, summarizing, and enhancing accessibility of existing evidence [14]. We combined outcomes from various types of SRs and synthesized qualitative and quantitative evidence. This type of synthesis is useful in identifying existing e-learning interventions used by RNs in their workplace settings and in describing the range of outcomes of interest measured, documented, and informed by the Nursing Care Performance Framework (NCPF).

We used the Cochrane Collaboration methodology [15] and other relevant works in this domain [16,17] to structure and report the SRSRs.

### Nursing Care Performance Framework

We planned to use the NCPF to guide our synthesis. The NCPF is based on Henriksen et al’s work [18], which depicts a conception of nursing care as a complex, whole entity; this entity is encompassed by many interrelated and interdependent subsystems and components that are logically coordinated and oriented toward the achievement of optimal outcomes for patients [19]. The NCPF is a systemic and organizational model aimed to measure the performance of nurses in the health care system through a set of indicators sensitive to various aspects of nursing care. The rationale for using the NCPF was based on our previous work [20]. We conducted an SRSRs of the effects of information and communications technologies on nursing care. We then categorized these effects based on the following nursing care subsystems pertaining to the NCPF: nursing care structure (eg, nursing staff supply and profiles, work conditions, and nursing staff stability), nursing services (eg, professional practice environment, nursing processes, and interventions), and patient outcomes (eg, patient functional status and care safety). Our first intent in this current SRSRs was to use the NCPF for guidance and as a starting point for data extraction and analysis, while remaining open to the emergence of new data (ie, outcomes) that were not part of the framework. We expected to get a comprehensive portrait of how dimensions and indicators of nursing care, as developed in the NCPF, could be influenced by the use of e-learning interventions in a nursing CE context. In other words, we identified, from the NCPF, a pre-established range of possible outcomes and indicators related to nursing care, for which data would be sought in this SRSRs [11].

### Eligibility Criteria

The scope was formulated using the population, intervention, comparison, outcomes, and study design (PICOS) format [15,21]. Eligibility criteria are presented in Table 1.

**Table 1:** Eligibility criteria.

SR^a^ components	Inclusion criteria	Exclusion criteria
Population	RNs^b^, according to the professional legislation of each country	Undergraduate nursing students in an academic context
Intervention	E-learning (ie, use of electronic, digital, or mobile devices to support learning) used in a continuing education context	Any type of simulation with a physical mannequin
Comparison	Face-to-face learning, any other e-learning intervention, or blended learning	N/A^c^
Outcomes	Primary outcomes: effects of e-learning on nursing care, including (1) nursing resources (eg, working conditions, nursing staff supply, and staff maintenance) and (2) nursing services (eg, nurses’ practice environments, nursing processes and interventions, and professional satisfaction) Secondary outcomes: Effects of e-learning on patient outcomes (eg, patients’ empowerment, comfort, and quality of life)	N/A
Study design	Systematic qualitative, quantitative, and mixed-studies reviews published in French, English, or Spanish	Grey literature and non-SR, such as literature reviews

^a^SR: systematic review.

^b^RN: registered nurse.

^c^N/A: not applicable.

### Search Strategy and Selection Criteria

We searched for articles that were published between January 1, 2006, and January 26, 2017, in the following electronic databases: PubMed, Embase, Cumulative Index of Nursing and Allied Health Literature (CINAHL), and Joanna Briggs Institute (JBI). We updated the search strategy to include articles published between January 1, 2017, and November 11, 2018. The search strategy time frame was partially informed by the work of de Caro et al [5,12], who conducted an SRSRs on a similar topic. They performed search strategies on articles published between 2003 and 2013. Included SRs were published between 2008 and 2013. We extended our search strategy to include articles published from 2006 onward to capture SRs that could have been missed in previous work.

We developed a structured search strategy that was validated by a health information specialist. We used the thesaurus terms from each database and used free text to target the *title* and *abstract* fields. An example of the search strategy in PubMed has been presented elsewhere [11]. For the JBI database, we hand searched the whole database for relevant literature. We collected the results of each database search in EndNote reference manager, version X7.7.1 (Clarivate Analytics) and we removed duplicate citations. Furthermore, we hand searched for relevant SRs, contacted authors to find other relevant works in this domain, and consulted the reference lists of included SRs.

### Selection of Systematic Reviews

We used DistillerSR (Evidence Partners), a Web-based SR software, to perform the screening and selection of SRs as well as the data extraction. A group of three reviewers (GR, JPG, and EH) independently screened the title and abstract of the papers in order to assess their eligibility. Each paper was reviewed twice, by two of the three reviewers. When consensus was not reached, arbitration was done with the third review author who was not involved in the selection of a specific SR. After the first round of screening, we retrieved full-text copies of publications that met the pre-established inclusion criteria and we assessed them twice.

### Methodological Quality Assessment of Included Systematic Reviews

The methodological quality assessment is defined as the critical appraisal of each SR and the extent to which authors of each SR met the highest possible standards in conducting and reporting their research process. Methodological quality also refers to risk of bias (ie, deviations of findings from the truth); flaws in design, conduct, analysis, and/or reporting can be the cause of these deviations [17].

Methodological quality was done independently by two reviewers (GR and JBP) using two critical appraisal tools in order to cover a wider and complementary range of criteria: Assessment of Multiple Systematic Reviews (AMSTAR) 2 [22] and Risk Of Bias In Systematic Reviews (ROBIS) [23]. AMSTAR 2 is a 16-item instrument that provides detailed and comprehensive assessment of SRs that include randomized or nonrandomized studies of health care interventions. ROBIS contains 21 signaling questions divided into three phases:

1. The assessment of relevance (optional).
2. The identification of concerns with the review process, in which bias can be introduced from within four domains:
  - Study eligibility criteria.
  - Identification and selection of studies.
  - Data collection and study appraisal.
  - Synthesis and findings.
3. Overall judgment about risk.

These tools are best suited for quantitative SRs and were not designed for systematic qualitative and mixed-studies reviews. However, because there was no consensus on how to assess methodological quality of qualitative and mixed-studies reviews at the time we began the SRSRs, we used both tools: AMSTAR 2 and ROBIS. Any disagreements that arose between the reviewers during the methodological quality assessment process were resolved through discussion.

### Data Extraction

A team of three authors (GR, JPG, and EH) conducted data extraction. Each paper was extracted independently by two of the three reviewers. We extracted the following data: (1) general characteristics of the SRs (eg, purpose, type of SR, number of primary studies included, populations, and settings); (2) details about e-learning interventions, comparisons, and the use of theories (ie, in the development and evaluation processes); and (3) outcomes, including their nature (ie, qualitative and/or quantitative) and direction (ie, positive, negative, or no effect). We used the adapted version of the NCPF [19] as a guide to extract outcomes. The dual extraction of outcomes data is particularly important, since these data are directly used in synthesizing the evidence that informs the conclusions of the review [23].

As recommended by Higgins et al [24], all the steps mentioned before (ie, selection of SRs, methodological quality assessment, and data extraction) were done by two authors working independently in order to minimize the risk of making mistakes and of being influenced by a single person’s biases. In total, four authors were involved in performing these steps (GR, JPG, EH, and JBP).

### Data Synthesis

The first author (GR) performed a qualitative thematic synthesis using a data-based convergent synthesis design [25,26]. We qualified quantitative data by using a narrative synthesis to describe the effect of e-learning on nursing care. We transformed the numerical data in specific themes and subthemes. This approach in conducting the data synthesis was chosen considering the mixed nature of evidence (ie, qualitative and quantitative) and the exploratory lens of this SRSRs. Two reviewers (EH and JPG) were involved in validating the data synthesis.

### Overlap in Systematic Reviews

As mentioned in the protocol [11], one of the challenges encountered when conducting SRSRs is the identification of overlap in SRs [27-29] (ie, “when the primary studies included within the SRs had multiple related publications that were referenced differently across SRs” [29]). Authors of SRSRs need to closely examine the content of the SRs and their included primary studies to accurately assess the extent and nature of the overlap. The ways of managing overlap in SRs depend on the purpose of the SRSRs and the method of data analysis [17]. Pollock et al [17] suggest that when the purpose of the SRSRs is to present and describe the body of knowledge that comes from SRs, it may be appropriate to include the results of all relevant SRs, regardless of the overlap across primary studies. Considering the exploratory lens of our SRSRs and our intent to draw a broad picture of the effects of e-learning interventions used by nurses in a CE context, we created a citation matrix [17] (see Multimedia Appendix 1) to visually represent the overlap between primary studies within included SRs. The implications of these overlaps do not have consequences on, for example, overestimating the effects of e-learning interventions that would bias recommendations to use a specific intervention over the others. The purpose of this SRSRs is not prescriptive and is not intended to inform or guide decision making, policy, or practice recommendations.

### Deviation From the Protocol

**Figure 1:**
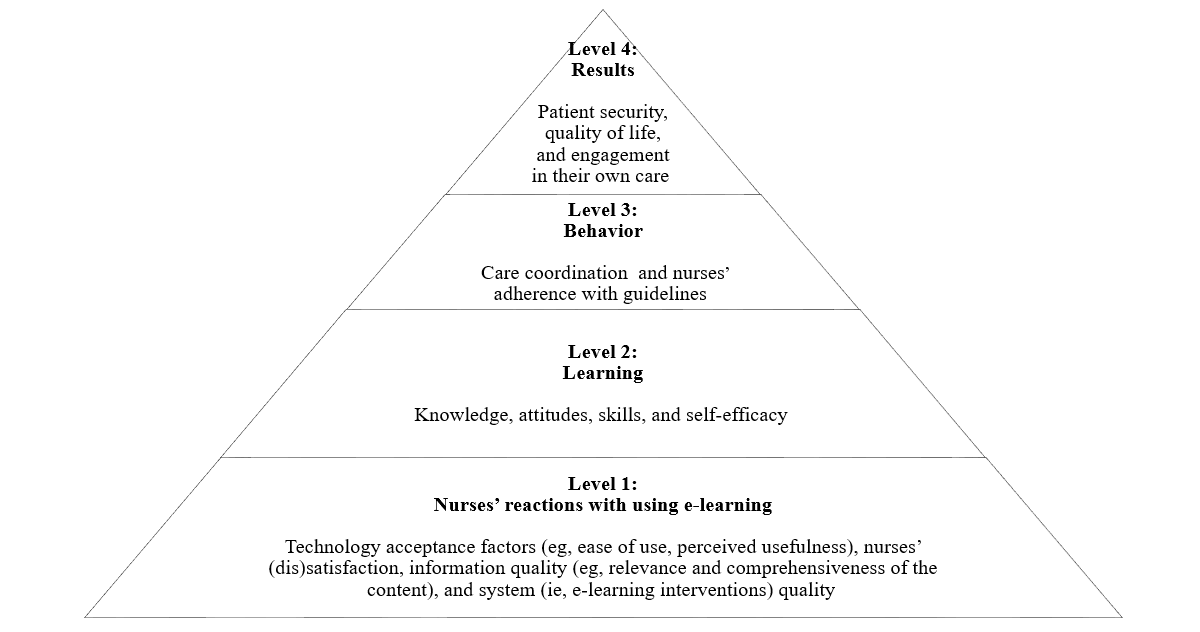
The Kirkpatrick model.

As previously mentioned, we used the NCPF to extract and classify the outcomes but we did not use it, as we had planned, to synthesize data regarding the effects of e-learning on nursing care. Indeed, this framework offers a broad perspective of the nursing care system, considering the diversity of nursing-sensitive indicators that are centered on structure and resources, nursing services and processes, and patient outcomes. However, most of these indicators do not reflect the current state of knowledge deriving from the effects of e-learning reported in the literature. These effects are rather circumscribed around nurses’ level of satisfaction, knowledge, or skills acquisition, which fit more with the Kirkpatrick model [30]. This model proposes four distinct levels as a sequence of ways to evaluate the effectiveness of an educational program: (1) reactions, (2) learning, (3) behavior, and (4) results. Level 1 (ie, reactions) is about nurses’ satisfaction with e-learning interventions. Level 2 (ie, learning) refers to the extent to which nurses change or improve attitudes, knowledge, skills, and/or self-efficacy as a result of attending the e-learning interventions [30]. Level 3 (ie, behavior) is the extent to which nurses’ learning has been translated into their postlearning behavior or their clinical performance [9]. Level 4 (ie, results) can be seen as patients’ health outcomes resulting from the influence of e-learning interventions on nurses’ behavior changes, which was adapted from Légaré et al [9]; this level can also be seen as other outcomes, such as costs [30]. In Figure 1, the Kirkpatrick model is presented, supported by some concrete examples provided by Shen et al [31].

Other frameworks could have been selected to extract and synthesize data, including the Expanded Outcomes Framework [32] or the Jeffries simulation model [33], since the *outcomes* component of the latter model can be potentially applicable to e-learning. However, we chose the Kirkpatrick model because it is well documented and extensively used in many educational contexts, including e-learning in the CE context [31,34].

Once we performed the first extraction using the NCPF, the data were read several times by three authors (GR, EH, and JPG). The first author built a thematic tree based on the reading of all material through line by line coding (ie, the inductive part) and based on existing works [19,30] (ie, the deductive part). This SRSRs was then guided by these two models [19,30] at different points in time: the use of the NCPF [19] was preplanned, and the use of the Kirkpatrick model [30] was decided during the process of data analysis and synthesis. The presentation of findings are supported by the four levels of evaluation [30].

Finally, we did not calculate the corrected covered area [27] in order to measure the actual degree of overlap in the SRSRs. We simply illustrated the overlap in a matrix (see Multimedia Appendix 1), as explained earlier.

## Results

### Search Results and Eligibility of Systematic Reviews

**Figure 2:**
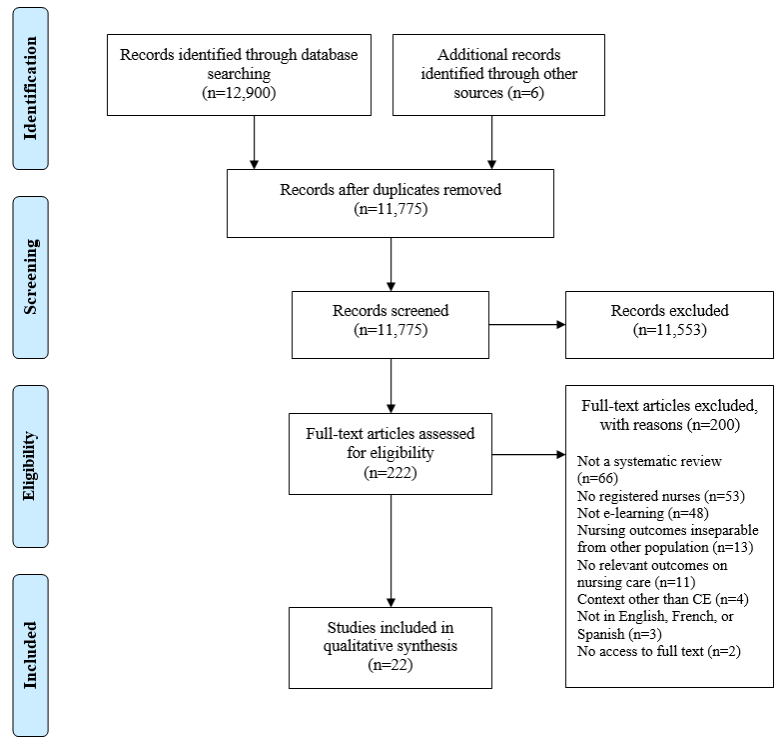
Preferred Reporting Items for Systematic Reviews and Meta-Analyses (PRISMA) flowchart. CE: continuing education.

The overall process of SR selection is illustrated with the Preferred Reporting Items for Systematic Reviews and Meta-Analyses (PRISMA) flow diagram [35] (see Figure 2). We identified a total of 12,906 records. After removing duplicate references, we assessed 11,775 records for eligibility. We retrieved 222 full-text papers for detailed evaluation, from which 22 SRs published between 2008 and 2018 met the eligibility criteria. The list of included SRs is presented in Multimedia Appendix 2. In Multimedia Appendix 3, we provide the references of excluded papers as well as the reasons for exclusion.

### Methodological Quality Assessment Results

**Figure 3:**
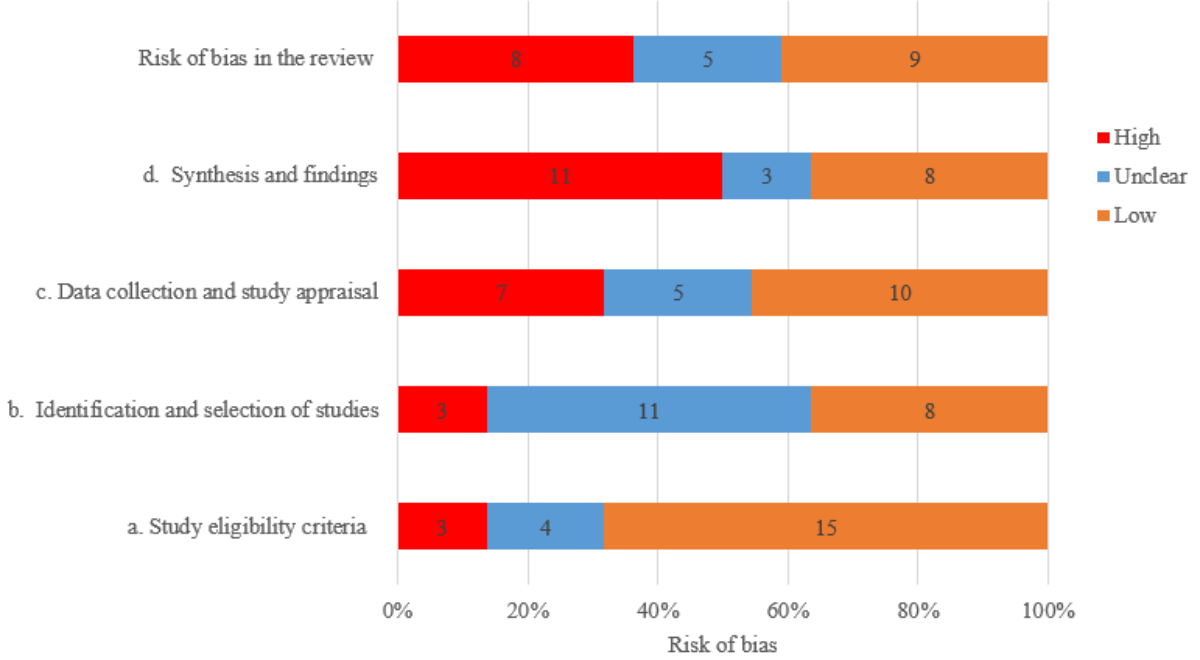
Methodological quality using the Risk Of Bias In Systematic Reviews (ROBIS) tool. The total risk of bias and the four domains of bias are shown. The numbers within the bars represent the number of systematic reviews.

**Figure 4:**
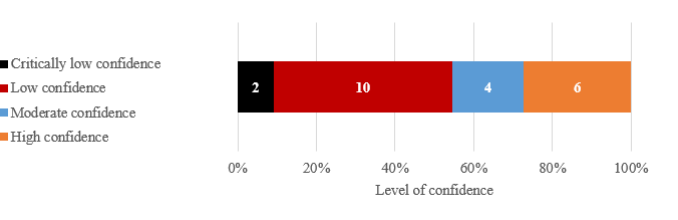
Methodological quality using the Assessment of Multiple Systematic Reviews (AMSTAR) 2. The numbers within the bars represent the number of systematic reviews.

We did not exclude papers based on methodological grounds, considering the scope of the SRSRs, which was not intended to inform action or decision making in terms of the most effective e-learning to impact nursing care. The assessment of methodological quality is presented individually for each SR (see Table 2) and globally (ie, all included SRs) using ROBIS (see Figure 3) and AMSTAR 2 (see Figure 4). Out of 22 SRs, 9 (41%) were at low risk of bias, 8 (36%) were at high risk of bias, and 5 (23%) had an unclear risk of bias. The assessment with AMSTAR 2 yielded the following results: out of 22 SRs, 6 (27%) had a high level of confidence, 4 (18%) had a moderate level of confidence, 10 (45%) had a low level of confidence, and 2 (9%) had a critically low level of confidence. The findings regarding the risk of bias and the level of confidence for the same SR were consistent across the two tools. For example, an SR [36] at high risk of bias according to the ROBIS tool was rated as having a low level of confidence using the AMSTAR 2.

**Table 2:** Methodological quality assessment for each individual SR^a^ included in this study using a combination of the ROBIS^b^ tool and the AMSTAR^c^ 2.

Author, year (type of SR)	Risk of bias using the ROBIS tool, Phase 2^d^: Identifying concerns with the review process—the four domains of bias				Risk of bias using the ROBIS tool, Phase 3: Judging overall risk of bias in the review	Level of confidence using the AMSTAR 2: final judgment
	Study eligibility criteria	Identification and selection of studies	Data collection and study appraisal	Synthesis and findings		
Bloomfield, 2008 [36] (QT^e^)	Low	High	High	High	High	Low
Brunero, 2012 [37] (MSR^f^)	Low	Unclear	Unclear	High	Unclear	Low
Byrne, 2008 [38] (QT)	High	Unclear	High	High	Unclear	Low
Carroll, 2009 [39] (MSR)	High	Unclear	High	High	High	Critically low
Chipps, 2012 [40] (QT)	Low	Low	Low	Unclear	Low	Moderate
Coyne, 2018 [41] (MSR)	Low	Low	Low	Unclear	Unclear	Moderate
Du, 2013 [42] (QT)	Low	Low	Low	Low	Low	High
Feng, 2013 [43] (QT)	Low	Unclear	Low	Low	Low	High
Freire, 2013 [44] (MSR)	Unclear	Low	High	High	High	Low
Harkanen, 2016 [45] (QT)	Low	Low	Low	Low	Low	High
Hegland, 2017 [46] (QT)	Low	Low	Low	Low	Low	High
Hines, 2015 [47] (QT)	Low	Low	Unclear	Low	Low	Moderate
Kakushi, 2016 [48] (MSR)	Unclear	Unclear	High	High	High	Low
Kang, 2017 [49] (QT)	Low	Unclear	Low	Low	Low	High
Knapp, 2008 [10] (MSR)	High	High	High	High	High	Critically low
Lahti, 2014 [1] (QT)	Low	Unclear	Low	Low	Low	Moderate
Lam-Antoniades, 2009 [50] (QT)	Low	Unclear	Unclear	High	High	Low
Lawn, 2017 [51] (MSR)	Unclear	High	Low	High	Unclear	Low
Nicoll, 2018 [3] (MSR)	Low	Unclear	Unclear	High	High	Low
Philips, 2012 [52] (MSR)	Unclear	Unclear	Unclear	Unclear	Unclear	Low
Sinclair, 2016 [4] (QT)	Low	Low	Low	Low	Low	High
Tomlinson, 2013 [53] (QT)	Low	Unclear	High	High	High	Low

^a^SR: systematic review.

^b^ROBIS: Risk Of Bias In Systematic Reviews.

^c^AMSTAR: Assessment of Multiple Systematic Reviews.

^d^Phase 1 is optional and consists of assessing the relevance of SRs. It was not performed nor described.

^e^QT: quantitative review.

^f^MSR: mixed-studies review.

### General Characteristics of Systematic Reviews and Participants

General characteristics of included SRs are shown in Multimedia Appendix 4. The first authors of the included SRs were from various countries: Australia (n=7), United Kingdom (n=4), Brazil (n=2), South Africa (n=1), China (n=1), Taiwan (n=1), Korea (n=1), Finland (n=2), Norway (n=1), United States (n=1), and Canada (n=1). In 8 SRs out of 22 (36%), there was an overlap regarding primary studies (see Multimedia Appendix 1).

We included any SRs that contained one or many primary studies focusing on RNs using e-learning interventions in a CE context, which means that SRs with populations other than RNs (eg, nursing students and other health care providers) were included as long as information about RNs was clearly retrievable. The ratio of primary studies targeting nurses in a CE context to the total number of primary studies pertaining to an SR was very low. For example, from Brunero et al [37], we extracted only 2 out of the 25 (8%) primary studies that met all of the eligibility criteria. Only 1 SR [10] included all primary studies (n=5) that concerned the population of interest (ie, RNs), the e-learning intervention, the outcomes of interest, and the CE context. When reported, the number of RNs across the SRs varied from 15 [53] to 658 [3]. RNs had different job titles (eg, nurse specialists, practice nurses, community nurses, and school nurses) and worked in different settings (eg, intensive care units, emergency departments, coronary critical care, medical-surgical, pediatrics, mental health, palliative care, geriatric hospitals, and primary care).

### E-Learning Interventions and Comparison Groups

There were a variety of e-learning interventions targeting nurses in a CE context (see Multimedia Appendix 5). Some examples are an online learning module regarding the use of brief motivational interviewing as a communication style to influence health behavior change [51] and online and interactive CD-ROM programs on medication administration skills and safety [45]. Other e-learning interventions were presented in terms of configuration, such as computer-assisted instructions [36], computer-based simulation [38], videoconferencing [53,52], and situated e-learning [43], while few had details on the instructional method, such as case-based learning [37].

Examples of comparison interventions included the following: electronic intervention, face-to-face intervention, no intervention, and blended learning. In four SRs [3,40,47,50], information about the theories or models used was reported regarding the engagement of stakeholders and the development or evaluation of e-learning interventions. These theories and models included engagement models [54,55], adult learning theory [56], the Kirkpatrick model [30], and the effects of information systems quality on nurses’ acceptance of the e-learning system [57].

### Effects of E-Learning

#### Overview

The outcomes are presented under different formats. First, the findings are grouped per systematic review along with a description of the interventions and the comparisons (see Multimedia Appendix 5). Second, they are described under a frequency table (see Table 3). Overall, positive outcomes (ie, effects reported in favor of e-learning interventions) are overrepresented compared to negative outcomes and those with no effect. Finally, the outcomes are synthesized and presented narratively under four main themes, informed by the Kirkpatrick model [30].

**Table 3:** Frequency and direction of outcomes.

Levels of evaluation from the Kirkpatrick model and subthemes			Number of documented outcomes from primary studies and direction of the effect			
			Negative	No effect	Positive	Total
**1. Nurse reactions with e-learning (n=11 SRs^a^)**						
	Total		7	0	27	34
	General		0	0	9	9
	Anonymity		0	0	1	1
	Authentic scenario		0	0	1	1
	Computer and internet experience		1	0	0	1
	Confidence in e-learning		0	0	1	1
	Content		0	0	1	1
	Discussion		0	0	1	1
	Information sharing		1	0	0	1
	Interactions		2	0	2	4
	Learners' experience		0	0	1	1
	Overall satisfaction		0	0	1	1
	Person-centered approach		0	0	1	1
	Satisfaction with interactive case studies		0	0	2	2
	Scope of reflection		0	0	1	1
	Sense of belonging		0	0	1	1
	Technical support		0	0	1	1
	Technology characteristics		3	0	3	6
**2. Nurse learning (n=18 SRs)**						
	Total		3	10	40	53
	**Knowledge (n=13 SRs)**					
		Total	1	5	18	24
		General	0	3	6	9
		Acute Physiology and Chronic Health Evaluation III scoring system	0	0	1	1
		Arterial blood gas interpretation	0	0	1	1
		Assessment (ability of neurological function)	0	0	1	1
		Assessment (general)	0	0	1	1
		Emergency preparedness	0	0	1	1
		Hospital quality	0	0	1	1
		Intravenous injections	0	0	2	2
		Medication administration	0	0	1	1
		Medication calculation	1	0	1	2
		Neonatal care	0	0	1	1
		Pain, physical and psychological symptoms, and loss	0	1	0	1
		Palliative care	0	1	1	2
	**Attitude and self-efficacy (n=3 SRs)**					
		Total	0	0	4	4
		Confidence postintervention	0	0	1	1
		Perceived effectiveness of e-learning	0	0	1	1
		Personal and professional development	0	0	1	1
		Stress in nurse-patient relationship	0	0	1	1
		Self-efficacy (general)	0	0	1	1
	**Skills (n=10 SRs)**					
		Total	2	5	18	25
		General	0	0	4	4
		Assessment (depression)	0	0	1	1
		Cannulation	1	0	0	1
		Cardiopulmonary resuscitation-defibrillation	1	0	0	1
		Care practice changes	0	0	1	1
		Child abuse detection	0	0	1	1
		Communication	0	0	1	1
		Critical appraisal of research literature	0	1	1	2
		Emergency preparedness skills performance	0	0	1	1
		Intravenous injections	0	1	0	1
		Medication preparation and administration	0	3	3	6
		Motivational interviewing	0	0	1	1
		Monitoring	0	0	1	1
		Neonatal care	0	0	1	1
		Universal precautions-related behaviors	0	0	1	1
		Scheduling activities	0	0	1	1
3. Behavior (change in practice) (n=0 SRs)			0	0	0	0
**4. Results (n=2 SRs)**						
	Total		0	0	1	1
	**Patient outcomes (n=1 SR)**					
		Nurses’ perceptions of care for older adults	0	0	1	1
	Cost (n=2 SRs)		0	0	2	2
Total			10	10	70	90

^a^SR: systematic review.

#### Level 1: Nurse Reactions With E-Learning Interventions

Nurse reactions with e-learning interventions have been described in 11 of the 22 SRs (50%) [3,10,36,39-42,44,50,51,52].

Positive outcomes were described in 8 out of 22 SRs (36%) [10,36,39,40,42,44,48,51], mostly in terms of nurse satisfaction with using e-learning for the following reasons: quality of content [44], importance of social interactions [40,48], active learning [48], flexibility [10,51], effectiveness and convenience of the technology, as well as quality of support received [10]. Other sources of nurse satisfaction were reported as follows: patient-centered approach, time-saving, and self-directed learning. Nurses stressed the importance of authentic scenarios and of practicing skills in the work context [51]. Nurses found higher satisfaction with e-learning than from videotaped courses [42], while in the SR by Lam-Antoniades et al [50], nurses found that there were advantages of e-continuing education over lecture courses. Otherwise, nurses felt satisfied with both e-learning programs and traditional in-classroom programs [3].

In 3 out of 22 SRs (14%), nurse dissatisfaction with e-learning interventions was explained by the following reasons: technical difficulties [10,40], a lack of computer experience and internet literacy, slower information exchange [10], and a preference for face-to-face format [52]. In one SR [51], nurses identified access, navigation, and time as challenges.

#### Level 2: Nurse Learning

##### Overview

Nurse learning outcomes were reported in 18 of 22 SRs (82%) [1,3,4,10,36-38,40-47,49,51]. We divided learning into three subthemes: knowledge, attitude and self-efficacy, and skills.

##### Knowledge

In 13 SRs out of 22 (59%) [1,3,10,37,53,40-45,49,52], nurses improved their knowledge with the help of e-learning interventions on many topics, including assessment of ability of neurological function [42], medication administration and calculation [45], physiology and chronic health evaluation [10], arterial blood gas interpretation, intervention focusing on a rare disease [3], and palliative care [52]. With the help of e-learning, nurses improved their knowledge compared to no intervention [43]. However, nurse acquisition of knowledge in a classroom was superior to e-learning for drug dose calculations [45].

In 7 SRs out of 22 (32%) [10,36,53,40,46,49,52], no effects were reported on nurse knowledge. There were nonsignificant differences in knowledge scores on drug dose calculation between groups [46] and in learning effectiveness outcomes between the face-to-face versus videoconference formats [53,52]. The effect size difference reported was not significant in these 2 SRs [10,49]. There were no significant outcomes related to learning on the topics of intravenous (IV) injections and medication administration and preparation [36]. No significant change was found in nurse knowledge related to pain, physical and psychological symptoms, and loss [40].

##### Attitude and Self-Efficacy

Higher self-efficacy and performance scores were generally found among nurses using the e-learning intervention [42]. Nurses had positive attitudes toward effectiveness of online learning modules for motivational interviewing [51]. They perceived benefits of e-learning on their personal and professional development [52]. Other nurses improved their confidence in reducing stress in the nurse-patient relationship [37].

##### Skills

In 9 SRs out of 22 (41%) [3,4,36,37,42-44,46,47], positives outcomes were documented related to the increase of skills following nurses’ participation in e-learning.

Nurses had better performance outcomes with e-learning compared to no intervention [42,43]. Nurses improved their skills after attending a 1-hour, e-learning-based, mental health education program on self-harm, demanding behavior, manipulation, and splitting and attention-seeking behavior [37]. These nurses also had positive comments regarding assessment, monitoring, communication, and interventions such as scheduling pleasant activities [37]. Furthermore, they rated items highly that were related to the extent to which training changed their care practices [37].

Nurses using e-learning interventions experienced positive outcomes related to universal precautions, IV injections, and medication administration [36,45]. The meta-analysis on computer-based simulation compared to other learning strategies showed significant effect in favor of e-learning for medication administration and preparation [46]. Nurses’ perceived skills in performing, and clinical use of, brief motivational interviewing were more favorable postintervention [3]. Nurses had better emergency preparedness as a result of e-learning than with no intervention and they improved child abuse detection with e-learning compared to no intervention [4]. An increase in nurses’ skills scores with e-learning related to neonatal care has been reported [44]. In terms of cognitive skills, nurses self-assessed their critical appraisal competencies positively regarding research literacy [47].

Negative outcomes were reported in 2 out of 22 SRs (9%). Cardiopulmonary resuscitation-defibrillation and defibrillation performance was worse among nurses using long-distance learning than that of the control group [42]. Nurses using the computer-based simulation to cannulate a real patient with force feedback had lower success at the first attempt.

Finally, 2 SRs out of 22 (9%) reported no effect by e-learning on skills. Nurses found no improvement in one critical appraisal competency related to research literacy: the identification of the sample [47]. *Core 2* errors related to preparation and administration of medication increased but the rate was not significant, as underlined in Bloomfield et al’s SR [36].

#### Level 3: Behavior

No outcomes related to nurses’ changes in practice were reported.

#### Level 4: Results

##### Patient Outcomes

In 1 of 22 SRs (5%), a positive outcome related to nurses’ perceptions of care outcomes for older adults was reported [37].

##### Cost

In 2 SRs out of 22 (9%) [10,37], positive outcomes were reported in terms of using intranet- and CD-ROM-based education as a low-cost method of providing education for nursing staff.

## Discussion

### Principal Findings

Our SRSRs aimed at synthesizing qualitative and quantitative evidence regarding the effects of e-learning interventions on nursing care in a CE context. To the best of our knowledge, this is the first broad synthesis on the impact of e-learning on nurses in a CE context. As we expected, heterogeneity was found between populations (ie, RNs and workplace settings), interventions, comparisons, outcomes, types of SRs, and corresponding evidence. Conducting a meta-analysis was not the purpose of this SRSRs.

### Main Outcomes: Four Levels of Evaluation

The most reported outcomes were learning (18/22, 82%), corresponding to Kirkpatrick’s evaluation level 2. Nurse skills were the most frequently reported, followed by knowledge. Outcomes related to evaluation level 1 (ie, nurses’ reactions with e-learning) were found in 11 out of the 22 SRs (50%). Authors of SRs described these reactions mainly with respect to technology characteristics, including perceived advantages and disadvantages (eg, navigability, technical difficulties, access, and flexibility). We found no SRs that reported outcomes regarding the translation of the content of e-learning interventions into nurses’ practice and behavior (ie, evaluation level 3). This finding does not mean that e-learning had no outcomes on practice. During the data analysis and interpretation, we used a conservative approach to classify the outcomes. Limited granularity of reported details is a well-known issue for authors of SRSRs and was observed in the included SRs. Therefore, it was difficult to know if skills, for example, improved nurses’ knowledge of medication administration and preparation or if it changed nurses’ practice. Only 1 SR included nurses’ perceptions of patients’ outcomes (ie, evaluation level 4) regarding care of elders; it also included 2 outcomes about costs. Overall, most reported outcomes were positive (n=70) as compared to negative (n=10) and neutral ones (n=10). This could indicate the presence of a reporting bias at the level of primary studies and SRs because of the disproportionate number of positive results [58].

Our findings related to the overrepresentation of the effects of e-learning interventions on reactions and learning, as well as the underrepresentation on practice and patient outcomes, are similar to those found in the literature among health care students, including nursing students [7,59]; physicians [9,60,61]; allied health practitioners [62]; and various health care providers [2]. However, Militello et al [34] conducted an SRSRs on the efficacy of computer-mediated continuing education for health care providers, including nurses, and they performed a meta-analysis. They classified their outcomes according to the Kirkpatrick model [30]. They found that 8 of the 11 SRs included measures of learner satisfaction (Level 1), 10 SRs included learning outcomes (Level 2), 9 included outcomes on provider behavior or performance (Level 3), and 5 included health and patient outcomes (Level 4). We can suppose that Militello et al [34] were more inclusive in their way of classifying outcomes related to practice change than we were in our data analysis and synthesis. Furthermore, many authors (eg, Légaré et al [9], Légaré et al [63], and Kitto et al [64]) are interested by this transition from Level 2 to Level 3 that can occur as a result of changes promoted by the content and format of continuing professional development activities, as well as how competencies are acquired and assessed. This transition not only depends on the acquisition of knowledge and skills, but also on a myriad of other elements related, for instance, to the intervention (eg, relative advantage), the outer context (eg, resources), the inner context (eg, organizational culture), individual characteristics (eg, learning style), and process (eg, planning) [65].

### Methodological Quality

The methodological quality of SRs varied greatly: 59% of SRs (13/22) had an overall high or unclear risk of bias, while 55% (12/22) had a low or critically low level of confidence. Only 41% (9/22) of SRs were assessed with low risk of bias while 45% (12/22) had a moderate or high level of confidence. Our results are different from those of Militello et al [34], who synthesized the methodological quality of SRs (n=11) on computer-mediated CE for health care providers. They used 11 items from the AMSTAR [66]. Out of 11 SRs, 5 were of moderate quality and 6 were of high quality. The authors only included quantitative SRs and meta-analyses.

These findings might be explained by several reasons. We used two tools that have been designed to assess systematic quantitative reviews. When we started this SRSRs in 2017 [11], no tool was available to appraise the quality of qualitative and mixed-studies reviews. Some criteria from the ROBIS tool and the AMSTAR 2, as well as their corresponding vocabulary (eg, meta-analysis, heterogeneity, and risk of bias), were not adapted to fit with the specificities of qualitative and mixed-studies reviews. Furthermore, the systematic methodology of some included SRs was not obvious. Some authors (eg, Knapp and Byers [10] and Carroll et al [39]) mentioned the word *systematic* in their paper but they did not provide all the details to fully explain the systematic nature of their work. It is important to highlight that methodological quality is one of the three dimensions of *quality* [67]. However, methodological quality is only one dimension of critical appraisal that could be performed and it is centered on how the SR is conducted [67]. It does not capture other concepts, such as the social relevance of findings and the applicability and transferability of findings to other contexts. The dimension of conceptual clarity can also be appraised and it is related to insightfulness, including the clarity, richness, and depth of description of a phenomenon [68]. Campbell et al [69] observed that methodological and conceptual quality can be inversely correlated. It means that papers that are appraised with a low methodological quality score are usually those providing good conceptual insight. This can be partially explained by the inadequacy regarding the reporting of qualitative research methods. We recommend appreciating the richness of our findings as a means to get a broad picture of the effects of e-learning interventions on nursing care. However, our results must be interpreted with caution and are not meant to guide or inform practice, nor are they meant to determine which e-learning interventions are better in supporting CE for nurses.

### Strengths and Potential Biases in the Systematic Review of Systematic Reviews Process

We used a comprehensive and systematic process throughout all stages of this SRSRs. In the search strategy, we used general keywords to explore the e-learning concept as an umbrella term, such as *virtual learning environment*, *distance learning*, *Web-based learning*, *e-learning*, and *m-learning*, among others. However, we did not use all specific key terms representing all forms of digital education, such as *serious games and gamification interventions*, *massive open online course*, *virtual reality*, and *virtual patient* [70]. Recent publications focused, for example, on serious games [59,71] and virtual reality [72], either in a context of preregistration training in health students or postregistration training among health care professionals such as nurses. Nonetheless, the use of general key terms allowed for the coverage of a wide range of potentially relevant references, considering the initial 12,906 records screened.

During the screening of titles, abstracts, and full texts, we observed that information regarding the population was sometimes misleading or incomplete, such as a population of “health students” (eg, Coyne et al [41]). In that case, instead of presuming that this abstract was not eligible based on the population, we decided to retrieve the full text. We discovered that nurses were targeted in some of these papers. Even if we were inclusive during the screening process, we may have excluded some references based on limited information provided in titles and/or abstracts. In order to limit the risk of excluding potentially relevant papers, we conducted the screening process as a team of three reviewers.

### Future Research

Our SRSRs targeted specific questions about the effects of e-learning interventions on nursing care in a CE context. Few details were provided regarding RN characteristics (eg, age and educational background) and interventions, including the SRs’ instructional designs. The lack of information granularity provided by the authors of SRs [69,70] is a limitation of conducting SRSRs. Cook [73] argued that these instructional designs can have an impact on the outcomes. Furthermore, few theoretical cues were given about active ingredients pertaining to the interventions that predict or explain professional or behavior change.

We would recommend using other types of knowledge synthesis to explore complementary and broader research questions. The following are some examples:

1. What are the contexts and mechanisms through which nurses and nursing students translate knowledge and skills from e-learning interventions to their practice and, consequently, how could they lead to specific outcomes among patients? How does it work? In that case, a realist review could be performed in a digital-based nursing education and CE context, with a lens similar to the work conducted by Wong et al [74].
2. How do nurses experience e-learning interventions in their work setting? How do they describe their impact on their practice or in their environment? A meta-synthesis of qualitative studies could be done to answer these questions.

It would be useful if authors of primary studies provided enough information regarding the intervention, the context, and mechanisms, including theoretical underpinnings, which could allow researchers to understand the components that can affect outcomes.

We would also suggest exploring other types of outcomes that can be related to having e-learning interventions in workplace settings. We are in agreement with Bernt et al [62], in that the relationship between access to continuing professional development and workforce retention is unknown. Other works could be done to investigate the influence of e-learning on nursing resources or structures [19], for instance, on nurse retention and working conditions.

Furthermore, most outcomes found in the literature focus on reactions and nurses’ satisfaction, learning, and change in practice. Change in knowledge and learning can be seen under a cognitivist learning approach. This approach targets the work of single individuals versus, for example, the social interactions that contribute to the learning experience of learners, seen under a social constructivist lens [3]. We would benefit from using a diversity of theoretical underpinnings, educational learning theories [75], and critical [76,77] and complexity theories [78] that have the potential to shed light on many perspectives (eg, individual, interpersonal, organizational, and sociopolitical) of envisioning education, professional development, and learners’ experience.

### Conclusions

The findings of this SRSRs show that the effects of e-learning are mainly reported in terms of reactions, knowledge, attitude, self-efficacy, and skills (ie, the first two evaluation levels from the Kirkpatrick model). The effectiveness of e-learning interventions used by nurses in a CE context remain unknown regarding how the learning can be transferred to change practice and affect patient outcomes. Further scientific, methodological, theoretical, and practice-based breakthroughs must feed the fast-growing field of e-learning in nursing education, especially in a life-learning perspective.

## Appendix

Multimedia Appendix 1 Overlap between primary studies within included systematic reviews.

Multimedia Appendix 2 List of included systematic reviews.

Multimedia Appendix 3 List of excluded papers.

Multimedia Appendix 4 General characteristics of included systematic reviews.

Multimedia Appendix 5 Interventions, comparisons, and outcomes from the studies.

## Abbreviations

AMSTAR: Assessment of Multiple Systematic Reviews
CE: continuing education
CIHR: Canadian Institutes of Health Research
CINAHL: Cumulative Index of Nursing and Allied Health Literature
FRQS: Fonds de recherche du Québec Santé
IV: intravenous
JBI: Joanna Briggs Institute
MSR: mixed-studies review
MSSS: Ministère de la Santé et des Services sociaux
NCPF: Nursing Care Performance Framework
PICOS: population, intervention, comparison, outcomes, and study design
PRISMA: Preferred Reporting Items for Systematic Reviews and Meta-Analyses
PROSPERO: International Prospective Register of Systematic Reviews
QT: quantitative review
RN: registered nurse
ROBIS: Risk Of Bias In Systematic Reviews
SPOR-SUPPORT: Strategy for Patient-Oriented Research—Support for People and Patient-Oriented Research and Trials
SR: systematic review
SRSRs: systematic review(s) of systematic reviews
