# Supplementary material for: Effects of E-Learning in a Continuing Education Context on Nursing Care: Systematic Review of Systematic Qualitative, Quantitative, and Mixed-Studies Reviews
Source: J Med Internet Res. 2019 Oct 2;21(10):e15118. doi: 10.2196/15118 (PMC6777280; doi:10.2196/15118)
Supplement: Multimedia Appendix 2 [file jmir_v21i10e15118_app2.pdf]

Multimedia Appendix 2: List of included systematic reviews.

1. Bloomfield JG, While AE, Roberts JD. Using computer assisted learning for clinical skills education in nursing: integrative review. J Adv Nurs 2008 Aug;63(3):222–235. PMID:18702768

2. Brunero S, Jeon Y-H, Foster K. Mental health education programmes for generalist health professionals: an integrative review. Int J Ment Health Nurs 2012 Oct;21(5):428–444. PMID:22500589

3. Byrne AJ, Pugsley L, Hashem MA. Review of comparative studies of clinical skills training. Med Teach 2008;30(8):764–767. PMID:18946819

4. Carroll C, Booth A, Papaioannou D, Sutton A, Wong R. UK health-care professionals' experience of on-line learning techniques: A systematic review of qualitative data. J Contin Educ Health Prof 2009;29(4):235–241. [doi: 10.1002/chp.20041]

5. Chipps J, Brysiewicz P, Mars M. A systematic review of the effectiveness of videoconference-based tele-education for medical and nursing education. Worldviews Evid Based Nurs 2012 Apr;9(2):78–87. PMID:22409341

6. Coyne E, Rands H, Frommolt V, Kain V, Plugge M, Mitchell M. Investigation of blended learning video resources to teach health students clinical skills: An integrative review. Nurse Educ Today 2018;63:101–107. [doi: 10.1016/j.nedt.2018.01.021]

7. Du S, Liu Z, Liu S, Yin H, Xu G, Zhang H, Wang A. Web-based distance learning for nurse education: a systematic review. Int Nurs Rev 2013 Jun;60(2):167–177. PMID:23691999

8. Feng J-Y, Chang Y-T, Chang H-Y, Erdley WS, Lin C-H, Chang Y-J. Systematic review of effectiveness of situated e-learning on medical and nursing education. Worldviews Evid Based Nurs 2013 Aug;10(3):174–183. PMID:23510119

9. Freire LM, Paula MA de, Duarte ED, Bueno M. Distance education in neonatal nursing scenarios: a systematic review. Rev Esc Enferm USP 2015 Jun;49(3):515–521. PMID:26107714

10. Härkänen M, Voutilainen A, Turunen E, Vehviläinen-Julkunen K. Systematic review and meta-analysis of educational interventions designed to improve medication administration skills and safety of registered nurses. Nurse Educ Today 2016 Jun;41:36–43. PMID:27138480

11. Hegland PA, Aarlie H, Strømme H, Jamtvedt G. Simulation-based training for nurses: Systematic review and meta-analysis. Nurse Educ Today 2017 Jul 1;54:6–20. [doi: 10.1016/j.nedt.2017.04.004]

12. Hines S, Ramsbotham J, Coyer F. The Effectiveness of Interventions for Improving the Research Literacy of Nurses: A Systematic Review. Worldviews Evid Based Nurs 2015 Oct;12(5):265–272. PMID:26422084

13. Kakushi LE, Évora YDM. Social networking in nursing education: integrative literature review. Rev Lat Am Enfermagem 2016;24:e2709. PMID:27384465

14. Kang J, Seomun G. Evaluating Web-Based Nursing Education's Effects: A Systematic Review and Meta-Analysis. West J Nurs Res 2017 Sep 6;0193945917729160. [doi: 10.1177/0193945917729160]

15. Knapp SJ, Byers JF. Use of the internet in staff development and its application in helping critical care nurses to lower family stress. J Nurses Staff Dev 2008 Feb;24(1):E1-8. PMID:18349757

16. Lahti M, Hätönen H, Välimäki M. Impact of e-learning on nurses' and student nurses knowledge, skills, and satisfaction: A systematic review and meta-analysis. Int J Nurs Stud 2014;51(1):136–149. [doi: 10.1016/j.ijnurstu.2012.12.017]

17. Lam-Antoniades M, Ratnapalan S, Tait G. Electronic continuing education in the health professions: an update on evidence from RCTs. J Contin Educ Health Prof 2009;29(1):44–51. PMID:19288566

18. Lawn S, Zhi X, Morello A. An integrative review of e-learning in the delivery of self-management support training for health professionals. BMC Med Educ 2017;17(1):183. PMID:29017521

19. Nicoll P, MacRury S, Woerden HC van, Smyth K. Evaluation of Technology-Enhanced Learning Programs for Health Care Professionals: Systematic Review. J Med Internet Res 2018;20(4):e131. [doi: 10.2196/jmir.9085]

20. Phillips JL, Piza M, Ingham J. Continuing professional development programmes for rural nurses involved in palliative care delivery: An integrative review. Nurse Educ Today 2012 May;32(4):385–392. [doi: 10.1016/j.nedt.2011.05.005]

21. Sinclair PM, Kable A, Levett-Jones T, Booth D. The effectiveness of Internet-based e-learning on clinician behaviour and patient outcomes: A systematic review. Int J Nurs Stud 2016;57:70–81. [doi: 10.1016/j.ijnurstu.2016.01.011]

22. Tomlinson J, Shaw T, Munro A, Johnson R, Madden DL, Phillips R, McGregor D. How does tele-learning compare with other forms of education delivery? A systematic review of tele-learning educational outcomes for health professionals. N S W Public Health Bull 2013 Nov;24(2):70–75. PMID:24195848
