# Supplementary material for: Effects of E-Learning in a Continuing Education Context on Nursing Care: Systematic Review of Systematic Qualitative, Quantitative, and Mixed-Studies Reviews
Source: J Med Internet Res. 2019 Oct 2;21(10):e15118. doi: 10.2196/15118 (PMC6777280; doi:10.2196/15118)
Supplement: Multimedia Appendix 3 [file jmir_v21i10e15118_app3.pdf]

Multimedia Appendix 3: List of excluded papers and reasons.

Not the right type of paper (n=66)

1. Aebersold M, Tschannen D. Simulation in nursing practice: the impact on patient care. Online journal of issues in nursing. 2013;18(2):6.
2. Aggarwal R, Darzi A, Grantcharov TP. Re: A systematic review of skills transfer after surgical simulation training. Annals of surgery. 2008 Oct;248(4):690-691; author reply 691. 18936587
3. Anderson C. How does social networking enhance the nursing narrative? Nursing management. 2009;40(9):16-20.
4. Anderson C, Brock T, Bates I, et al. Transforming health professional education. Am J Pharm Educ. 2011 Mar 10;75(2):22. 21519412
5. Bailey J. The age of virtual learning. Midwives. 2012;15(1):34-35.
6. Baker JD. Serious Games and Perioperative Nursing. AORN Journal. 2009;90(2):173-175.
7. Beard K, Morote ES. Using podcasts with narrative pedagogy: are learning objectives met? Nursing education perspectives. 2010;31(3):186-187.
8. Bera K, Biswas T, Biswas R. 'User Driven Health Care': Understanding the complexity of clinical experience through online 'conversational learning' networks. Australasian Medical Journal. 2012;5(1):60-61.
9. Brown JF. Applications of simulation technology in psychiatric mental health nursing education. Journal of Psychiatric and Mental Health Nursing. 2008;15(8):638-644.
10. Buckley KM, Rietschel M. Training and instructional strategies for the use of web conferencing in nursing education. Computers, informatics, nursing. 2012;30(11):569-576.
11. Campbell M, Themessl-Huber M, Mole L, Scarlett V. Using simulation to prepare students for interprofessional work in the community. The Journal of nursing education. 2007;46(7):340.
12. Carlton KH, Dillard N, Campbell BR, Baker NA. Personal digital assistants for classroom and clinical use. CIN - Computers Informatics Nursing. 2007;25(5):253-258.

13. Cates LA, Wilson D. Acquisition and maintenance of competencies through simulation for neonatal nurse practitioners. Advances in Neonatal Care. 2011;11(5):321-327.

14. Considine J, Brennan D. Effect of an evidence-based paediatric fever education program on emergency nurses' knowledge. Accident and Emergency Nursing. 2007;15(1):10-19.

15. Cooper S. Simulation versus lecture? Measuring educational impact: considerations for best practice. Evid Based Nurs. 2016 Apr;19(2):55. 26494851

16. Corbridge SJ, McLaughlin R, Tiffen J, et al. Using simulation to enhance knowledge and confidence. The Nurse practitioner. 2008;33(6):12-13.

17. Culley JM, Polyakova-Norwood V. Synchronous online role play for enhancing community, collaboration, and oral presentation proficiency. Nursing Education Perspectives. 2012;33(1):51-54.

18. Curran C, Sheets D, Kirkpatrick B, Bauldoff GS. Virtual patients support point-of-care nursing education. Nursing management. 2007;38(12):27-33.

19. Day L. Simulation and the teaching and learning of practice in critical care units. American journal of critical care : an official publication, American Association of Critical-Care Nurses. 2007;16(5):504-507.

20. De Caro W, Marucci AR, Giordani M, Sansoni J. [E-learning and university nursing education: an overview of reviews]. Prof Inferm. 2014 Apr-Jun;67(2):107-116. 25134508

21. De Fiore L. [E-learning and the continuing professional development in medicine]. Recenti Prog Med. 2010 Jun;101(6):251-252. 20672573

22. Elfrink VL, Harding T. Delivering online nursing education across the international dateline. Computers, informatics, nursing : CIN. 2008;26(5):249-254.

23. Eppich W, Howard V, Vozenilek J, Curran I. Simulation-based team training in healthcare. Simulation in healthcare : journal of the Society for Simulation in Healthcare. 2011;6 Suppl:S14-19.

24. Erdley WS, Hansen M. Overview of smart phone video essentials. CIN - Computers Informatics Nursing. 2012;30(3):119-122.

25. Farrell M. Nursing and midwifery education using mobile technologies. Australian nursing journal (July 1993). 2006;14(1):25.

26. Ferrell BR, Winn R. Medical and nursing education and training opportunities to improve survivorship care. Journal of clinical oncology : official journal of the American Society of Clinical Oncology. 2006 Nov 10;24(32):5142-5148. 17093277

27. Finkelstein J, Bedra M. Is Internet Search Better than Structured Instruction for Web-Based Health Education? International Conference on Informatics, Management, and Technology in Healthcare (ICIMTH) Conference, July 5-7th, Athens, Greece. Studies in Health Technology & Informatics. 2013;190:65-67. 104109704.

28. Finn GM, Scott L. Timely and tiny: supporting remote learning through the use of personal digital assistants. Medical education. 2011;45(5):521-522.

29. Forrest K, McKimm J. Using simulation in clinical education. British Journal of Hospital Medicine. 2010;71(6):345-349.

30. Gant LT. Human Simulation in Emergency Nursing Education: Current Status. Journal of Emergency Nursing. 2007;33(1):69-71.

31. George PP, Bhone MK, Saxena N, et al. Online e-learning for postregistration healthcare professionals-a bibliographic analysis of the literature. Annals of the Academy of Medicine Singapore. 2015;44(10):S460.

32. Greig S. Electronic online infection prevention and control modules for health care workers. Australian nursing journal (July 1993). 2011;19(2):41, 43.

33. Gruendemann BJ. Distance Learning and Perioperative Nursing. AORN Journal. 2007;85(3):574-576,578-586.

34. Hansen MM. Versatile, immersive, creative and dynamic virtual 3-D healthcare learning environments: a review of the literature. J Med Internet Res. 2008 Sep 01;10(3):e26. 18762473

35. Hantler A. Helping student nurses learn. Nursing New Zealand (Wellington, NZ : 1995). 2006;12(5):24-26.

36. Hao AT, Chang HK, Chong PP. Mobile learning for nursing education. AMIA Annual Symposium proceedings / AMIA Symposium AMIA Symposium. 2006:943.

37. Hopkins DD. The emergence of online learning in PN Education. The Journal of practical nursing. 2008;58(4):4-7.

38. Kaas MJ. Lessons Learned: Providing Access Through Distance Education. Journal of the American Psychiatric Nurses Association. 2012;18(3):192.

39. Krishnasamy C, Ong SY, Yock Y, et al. Factors influencing the implementation, adoption, use, sustainability and scalability of mLearning for medical and nursing education: A systematic review protocol. Systematic Reviews. 2016;5(1).

40. Labeau SO. Is there a place for e-learning in infection prevention? Aust Crit Care. 2013 Nov;26(4):167-172. PMID: 24183831

41. Logan R. Using YouTube in Perioperative Nursing Education. AORN Journal. 2012;95(4):474-481.

42. Long A, Kerfoot BP, Chopra S, Shaw T. Online spaced education to supplement live courses. Med Educ. 2010 May;44(5):519-520. 20374457

43. Marsden J. Online qualification for emergency nurses. Emergency nurse : the journal of the RCN Accident and Emergency Nursing Association. 2009;17(1):37.

44. Mason L. Using simulation to further best practices in nursing leadership. Critical care nurse. 2011;31(6):12.

45. Mayes P, Schott-Baer D. Professional development for night shift nurses. Journal of continuing education in nursing. 2010;41(1):17-22; quiz 23-24.

46. McFetrich J, Price C. Simulators and scenarios: Training nurses in emergency care. Medical education. 2006;40(11):1139.

47. Militello LK, Gance-Cleveland B, Aldrich H, Kamal R. A Methodological Quality Synthesis of Systematic Reviews on Computer-Mediated Continuing Education for Healthcare Providers. Worldviews on Evidence-Based Nursing. 2014;11(3):177-186. PMID: 103963365.

48. Nicoll LH. Nursing education enhanced by informatics. CIN - Computers Informatics Nursing. 2011;29(SUPPL. 6):TC81.

49. Pank CM. Online education. American Journal of Nursing. 2007;107(5):74-76.

50. Pereira SR, Loddi SA, Larangeira VA, Labrada L, Bandiera-Paiva P. Brazilian experiments in mobile learning for health professionals. 2013. Stud Health Technol Inform. 2013;190:160-2. PMID: 23823409

51. Peterson DS. A meta-analytic study of adult self-directed learning and online nursing education: a review of research from 1995 to 2007: Capella University; 2008. PhD dissertation.

52. Petty J. Interactive, technology-enhanced self-regulated learning tools in healthcare education: a literature review. Nurse Educ Today. 2013 Jan;33(1):53-59. 22818225

53. Phillippi JC, Wyatt TH. Smartphones in nursing education. CIN - Computers Informatics Nursing. 2011;29(8):449-454.

54. Phillips B, Shaw RJ, Sullivan DT, Johnson C. Using virtual environments to enhance nursing distance education. Creative nursing. 2010;16(3):132-135.

55. Saxena N, Bhone MK, Lim KTK, et al. Virtual reality environments for pre and postregistration health professional education. What's the evidence base? Annals of the Academy of Medicine Singapore. 2015;44(10):S457.

56. Schmitt TL, Sims-Giddens SS, Booth RG. Social media use in nursing education. Online journal of issues in nursing. 2012;17(3):2.

57. Schultz SJ. Evidence-based strategies for teaching dysrhythmia monitoring practices to staff nurses. Journal of continuing education in nursing. 2011;42(7):308-319.

58. Sherrod BC, Sherrod D. Is online education right for you? Nursing Management. 2013;44(4):40-45.

59. Southernwood J. Distance learning: the future of continuing professional development. Community practitioner : the journal of the Community Practitioners' & Health Visitors' Association. 2008;81(10):21-23.

60. Ulrich B. Using high fidelity patient simulation as an education tool in nephrology. Nephrology nursing journal : journal of the American Nephrology Nurses' Association. 2011;38(6):459.

61. Williams J, Lakhani N. E-learning for interprofessional education: a challenging option. Journal of interprofessional care. 2010;24(2):201-203.

62. Brunette V, Thibodeau-Jarry N. Simulation as a Tool to Ensure Competency and Quality of Care in the Cardiac Critical Care Unit. Canadian Journal of Cardiology 2017 Jan;33(1):119–127. [doi: 10.1016/j.cjca.2016.10.015]

63. Curran V, Matthews L, Fleet L, Simmons K, Gustafson DL, Wetsch L. A Review of Digital, Social, and Mobile Technologies in Health Professional Education. The Journal of continuing education in the health professions 2017 Summer;37(3):195–206. [doi: 10.1097/ceh.0000000000000168]

64. McCutcheon LRM, Alzghari SK, Lee YR, Long WG, Marquez R. Interprofessional education and distance education: A review and appraisal of the current literature. Currents in Pharmacy Teaching and Learning 2017 Jul;9(4):729–736. [doi: 10.1016/j.cptl.2017.03.011]

65. McLoughlin C, Patel KD, O'Callaghan T, Reeves S. The use of virtual communities of practice to improve interprofessional collaboration and education: findings from an integrated review. Journal of Interprofessional Care 2018 Mar 4;32(2):136–142. PMID:29161155

66. Tavares APC, Leite BS, Silveira IA, Santos TD dos, Brito W de AP de, Camacho ACLF. Analysis of Brazilian publications on distance education in nursing: integrative review. Revista Brasileira de Enfermagem 2018 Feb;71(1):214–222. [doi: 10.1590/0034-7167-2016-0454]

Not the good population (n=53)

1. Continuing medical education: Supplement 1, April 2011. An evidence-based systematic review on medical therapies for inflammatory bowel diseases. Am J Gastroenterol. 2011 Apr;106 Suppl 1:S1. 21472011

2. A systematic review investigating the use of Twitter and Facebook in university-based healthcare education. Health Education (0965-4283). 2014;114(5):347-366. 107832902. Language: English. Entry Date: 20141031. Revision Date: 20150712. Publication Type: Journal Article

3. Abdulmajed H, Park YS, Tekian A. Assessment of educational games for health professions: a systematic review of trends and outcomes. Medical teacher. 2015;37:S27-S32.

4. Al-Ghareeb AZ, Cooper SJ. Barriers and enablers to the use of high-fidelity patient simulation manikins in nurse education: an integrative review. Nurse Educ Today. 2016 Jan;36:281-286. 26323885

5. Alexandrou E, Ramjan L, Murphy J, et al. Training of Undergraduate Clinicians in Vascular Access: An Integrative Review. Journal of the Association for Vascular Access. 2012;17(3):146-158. 104158311. Language: English. Entry Date: 20131112. Revision Date: 20150818. Publication Type: Journal Article

6. Atlantis E, Cheema BS. Effect of audience response system technology on learning outcomes in health students and professionals: an updated systematic review. International journal of evidence-based healthcare. 2015;13(1):3-8.

7. Bearman M, Palermo C, Allen LM, Williams B. Learning Empathy Through Simulation: A Systematic Literature Review. Simulation in healthcare : journal of the Society for Simulation in Healthcare. 2015;10(5):308-319.

8. Bhana VM. Interpersonal skills development in Generation Y student nurses: a literature review. Nurse Educ Today. 2014 Dec;34(12):1430-1434. 24880789

9. Blakely G, Skirton H, Cooper S, Allum P, Nelmes P. Educational gaming in the health sciences: systematic review. J Adv Nurs. 2009 Feb;65(2):259-269. 19032512

10. Brulet A, Llorca G, Letrilliart L. Medical wikis dedicated to clinical practice: a systematic review. J Med Internet Res. 2015 Feb 19;17(2):e48. 25700482

11. Buckley S, Coleman J, Davison I, et al. The educational effects of portfolios on undergraduate student learning: a Best Evidence Medical Education (BEME) systematic review. BEME Guide No. 11. Med Teach. 2009 Apr;31(4):282-298. 19404891

12. Button D, Harrington A, Belan I. E-learning & information communication technology (ICT) in nursing education: A review of the literature. Nurse Educ Today. 2014 Oct;34(10):1311-1323. 23786869

13. Cant RP, Cooper SJ. Simulation-based learning in nurse education: systematic review. J Adv Nurs. 2010 Jan;66(1):3-15. 20423432

14. Cartledge P, Miller M, Phillips B. The use of social-networking sites in medical education. Med Teach. 2013 Oct;35(10):847-857. 23841681

15. Cohen NL, Carbone ET, Beffa-Negrini PA. The design, implementation, and evaluation of online credit nutrition courses: a systematic review. J Nutr Educ Behav. 2011 Mar-Apr;43(2):76-86. 21392711

16. Cook DA. How much evidence does it take? A cumulative meta-analysis of outcomes of simulation-based education. Med Educ. 2014 Aug;48(8):750-760. 25039731

17. De Gagne JC, Oh J, Kang J, Vorderstrasse AA, Johnson CM. Virtual worlds in nursing education: a synthesis of the literature. J Nurs Educ. 2013 Jul;52(7):391-396. 23755942

18. Doyle GJ, Garrett B, Currie LM. Integrating mobile devices into nursing curricula: opportunities for implementation using Rogers' Diffusion of Innovation model. Nurse Educ Today. 2014 May;34(5):775-782. 24268745

19. Fisher D, King L. An integrative literature review on preparing nursing students through simulation to recognize and respond to the deteriorating patient. J Adv Nurs. 2013 Nov;69(11):2375-2388. 23734570

20. French HP, Dowds J. An overview of Continuing Professional Development in physiotherapy. Physiotherapy. 2008;94(3):190-197.

21. Horne EM, Sandmann LR. Current trends in systematic program evaluation of online graduate nursing education: an integrative literature review. J Nurs Educ. 2012 Oct;51(10):570-576. 22909043

22. Irwin P, Coutts R. A Systematic Review of the Experience of Using Second Life in the Education of Undergraduate Nurses. J Nurs Educ. 2015 Oct;54(10):572-577. 26431517

23. Jayakumar N, Brunckhorst O, Dasgupta P, Khan MS, Ahmed K. e-Learning in Surgical Education: A Systematic Review. Journal of surgical education. 2015 Nov-Dec;72(6):1145-1157. 26111822

24. Jeffrey K, Bourgeois S. The effect of personal digital assistants in supporting the development of clinical reasoning in undergraduate nursing students: A systematic review. JBI Library of Systematic Reviews. 2015;9(2):38-68.

25. Jin J, Bridges SM. Educational technologies in problem-based learning in health sciences education: a systematic review. J Med Internet Res. 2014 Dec 10;16(12):e251. 25498126

26. Kind T, Evans Y. Social media for lifelong learning. International review of psychiatry (Abingdon, England). 2015 Apr;27(2):124-132. 25906988

27. Kleinert R, Wahba R, Chang DH, et al. 3D immersive patient simulators and their impact on learning success: a thematic review. J Med Internet Res. 2015 Apr 08;17(4):e91. 25858862

28. Koch LF. The nursing educator's role in e-learning: a literature review. Nurse Educ Today. 2014 Nov;34(11):1382-1387. 24797277

29. Lee J, Oh PJ. Effects of the Use of High-Fidelity Human Simulation in Nursing Education: A Meta-Analysis. J Nurs Educ. 2015 Sep;54(9):501-507. 26334336

30. Loke Jennifer CF. Computer mediated conferencing - A hope or hype for healthcare education in higher learning?: A review of the Literature. Nurse Education Today. 2007;27(4):318-324.

31. Mancuso JM. Perceptions of distance education among nursing faculty members in North America. Nurs Health Sci. 2009 Jun;11(2):194-205. 19519708

32. Mancuso-Murphy J. Distance education in nursing: an integrated review of online nursing students' experiences with technology-delivered instruction. The Journal of nursing education. 2007;46(6):252-260.

33. McCutcheon K, Lohan M, Traynor M, Martin D. A systematic review evaluating the impact of online or blended learning vs. face-to-face learning of clinical skills in undergraduate nurse education. Journal of advanced nursing. 2015;71(2):255-270.

34. Miller M, Jensen R. Avatars in nursing: an integrative review. Nurse Educ. 2014 Jan-Feb;39(1):38-41. 24300258

35. Murdoch NL, Bottorff JL, McCullough D. Simulation education approaches to enhance collaborative healthcare: a best practices review. Int J Nurs Educ Scholarsh. 2014 Jan 08;10. 24402885

36. Norman J. Systematic review of the literature on simulation in nursing education. Abnf j. 2012 Spring;23(2):24-28. 22774355

37. Patterson BJ, Krouse AM, Roy L. Student outcomes of distance learning in nursing education: an integrative review. Comput Inform Nurs. 2012 Sep;30(9):475-488. 22592452

38. Pittman OA. The use of simulation with advanced practice nursing students. Journal of the American Academy of Nurse Practitioners. 2012;24(9):516-520.

39. Raman J. Mobile technology in nursing education: where do we go from here? A review of the literature. Nurse Educ Today. 2015 May;35(5):663-672. 25665926

40. River J, Currie J, Crawford T, Betihavas V, Randall S. A systematic review examining the effectiveness of blending technology with team-based learning. Nurse Educ Today. 2016 Oct;45:185-192. 27541947

41. Strandell-Laine C, Stolt M, Leino-Kilpi H, Saarikoski M. Use of mobile devices in nursing student-nurse teacher cooperation during the clinical practicum: an integrative review. Nurse Educ Today. 2015 Mar;35(3):493-499. 25456259

42. Swift L. Online communities of practice and their role in educational development: A systematic appraisal. Community Practitioner. 2014;87(4):28-31.

43. Vincent MA, Sheriff S, Mellott S. The efficacy of high-fidelity simulation on psychomotor clinical performance improvement of undergraduate nursing students. Comput Inform Nurs. 2015 Feb;33(2):78-84. 25636043

44. Vitale E. Clinical teaching models for nursing practice: a review of literature. Prof Inferm. 2014 Apr-Jun;67(2):117-125. 25134509

45. Weaver A. High-fidelity patient simulation in nursing education: an integrative review. Nurs Educ Perspect. 2011 Jan-Feb;32(1):37-40. 21473481

46. Adib-Hajbaghery M, Sharifi N. Effect of simulation training on the development of nurses and nursing students' critical thinking: A systematic literature review. Nurse Education Today 2017 Mar;50:17–24. [doi: 10.1016/j.nedt.2016.12.011]

47. Berndt A, Murray CM, Kennedy K, Stanley MJ, Gilbert-Hunt S. Effectiveness of distance learning strategies for continuing professional development (CPD) for rural allied health practitioners: a systematic review. BMC Medical Education 2017 Dec;17(1):117. [doi: 10.1186/s12909-017-0949-5]

48. Breytenbach C, ten Ham-Baloyi W, Jordan PJ. An Integrative Literature Review of Evidence-Based Teaching Strategies for Nurse Educators: Nursing Education Perspectives 2017;38(4):193–197. [doi: 10.1097/01.NEP.0000000000000181]

49. Chen F, Lui AM, Martinelli SM. A systematic review of the effectiveness of flipped classrooms in medical education. Medical Education 2017 Jun 1;51(6):585–597. [doi: 10.1111/medu.13272]

50. Cho D, Cosimini M, Espinoza J. Podcasting in medical education: a review of the literature. Korean J Med Educ 2017 Dec;29(4):229–239. PMID:29207454

51. Voutilainen A, Saaranen T, Sormunen M. Conventional vs. e-learning in nursing education: A systematic review and meta-analysis. Nurse Education Today 2017 Mar;50:97–103. [doi: 10.1016/j.nedt.2016.12.020]

52. Webb L, Clough J, O'Reilly D, Wilmott D, Witham G. The utility and impact of information communication technology (ICT) for pre-registration nurse education: A narrative synthesis systematic review. Nurse Education Today 2017 Jan;48:160–171. [doi: 10.1016/j.nedt.2016.10.007]

53. Wu XV, Chan YS, Tan KHS, Wang W. A systematic review of online learning programs for nurse preceptors. Nurse Education Today 2018 Jan;60:11–22. [doi: 10.1016/j.nedt.2017.09.010]

Not the good intervention (n=48)

1. Adamson K. A Systematic Review of the Literature Related to the NLN/Jeffries Simulation Framework. *Nurs Educ Perspect*. 2015 Sep-Oct;36(5):281-291. 26521495

2. Akl EA, Kairouz VF, Sackett KM, et al. Educational games for health professionals. *The Cochrane database of systematic reviews*. 2013;3:CD006411.

3. Bellolio MF, Stead LG. Continuing Education Meetings and Workshops: Effects on Professional Practice and Health Care Outcomes. *Annals of Emergency Medicine*. 2009;53(5):685-687.

4. Bhoopathi PS, Sheoran R, Adams CE. Educational games for mental health professionals: a Cochrane review. *The international journal of psychiatric nursing research*. 2007;12(3):1497-1502.

5. Bloice MD, Simonic KM, Holzinger A. On the usage of health records for the design of Virtual Patients: a systematic review. *BMC Med Inform Decis Mak*. 2013 Sep 08;13:103. 24011027

6. Bogetz JF, Rassbach CE, Bereknyei S, et al. Training health care professionals for 21st-century practice: a systematic review of educational interventions on chronic care. *Acad Med*. 2015 Nov;90(11):1561-1572. 26039140

7. Bould MD, Boet S, Fung L, et al. Transfer of learning and patient outcome in simulated CRM. *Canadian Journal of Anesthesia*. 2014;61:S149.

8. Boulos MNK, Wheeler S. The emerging Web 2.0 social software: An enabling suite of sociable technologies in health and health care education. *Health Information and Libraries Journal*. 2007;24(1):2-23.

9. Brewer EP. Successful techniques for using human patient simulation in nursing education. *Journal of nursing scholarship : an official publication of Sigma Theta Tau International Honor Society of Nursing*. 2011 Sep;43(3):311-317. 21884377

10. Cook DA, Brydges R, Hamstra SJ, et al. Comparative effectiveness of technology-enhanced simulation versus other instructional methods: a systematic review and meta-analysis. *Simulation in healthcare : journal of the Society for Simulation in Healthcare*. 2012;7(5):308-320.

11. Cook DA, Brydges R, Zendejas B, Hamstra SJ, Hatala R. Mastery learning for health professionals using technology-enhanced simulation: A systematic review and meta-analysis. *Academic Medicine*. 2013;88(8):1178-1186.

12. Cook DA, Hatala R, Brydges R, et al. Technology-enhanced simulation for health professions education: a systematic review and meta-analysis. *Jama*. 2011 Sep 07;306(9):978-988. 21900138

13. Ding M, Metcalfe H, Gallagher O, Hamdorf JM. Evaluating trauma nursing education: An integrative literature review. *Nurse Educ Today*. 2016 Sep;44:33-42. 27429327

14. Elliott S, Murrell K, Harper P, Stephens T, Pellowe C. A comprehensive systematic review of the use of simulation in the continuing education and training of qualified medical, nursing and midwifery staff. *JBI Library of Systematic Reviews*. 2011;9(17):538-587.

15. Eunjoo J, Hyeoun-Ae P. Nursing Intervention using smartphone technologies; a systematic review and meta-analysis. *Studies in Health Technology & Informatics*. 2015;210:321-325. PMID: 109618854.

16. Franklin AE, Lee CS. Effectiveness of simulation for improvement in self-efficacy among novice nurses: a meta-analysis. *J Nurs Educ*. 2014 Nov 01;53(11):607-614. 25350902

17. Gunnarsdóttir S, Grétarsdóttir Eö. Systematic Review of Interventions Aimed at Nurses to Improve Pain Management. *Nordic Journal of Nursing Research & Clinical Studies / Vård i Norden*. 2011;31(4):16-21. 104518853.

18. Hallenbeck VJ. Use of high-fidelity simulation for staff education/development: a systematic review of the literature. *Journal for Nurses in Staff Development*. 2012;28(6):260-269; quiz E269. PMID: 107966054.

19. Harder BN. Use of simulation in teaching and learning in health sciences: a systematic review. *J Nurs Educ*. 2010 Jan;49(1):23-28. 19731886

20. Haywood H, Pain H, Ryan S, Adams J. Engagement with continuing professional development: development of a service model. *J Allied Health*. 2012 Summer;41(2):83-89. PMID: 22735821

21. Jansson M, KÃ¤Ã¤riÃ¤inen M, KyngÃ¤s H. Effectiveness of Simulation-Based Education in Critical Care Nurses' Continuing Education: A Systematic Review. *Clinical Simulation in Nursing*. 2013;9(9):e355-360. PMID: 104212037.

22. Lane C, Rollnick S. The use of simulated patients and role-play in communication skills training: a review of the literature to August 2005. *Patient Educ Couns*. 2007 Jul;67(1-2):13-20. 17493780

23. Levett-Jones T, Lapkin S. A systematic review of the effectiveness of simulation debriefing in health professional education. *Nurse Education Today*. 2014;34(6):e58-63. PMID: 103944437.

24. Liu WI, Edwards H, Courtney M. Review of continuing professional education in case management for nurses. *Nurse Education Today*. 2009;29(5):488-492.

25. Ma IW, Brindle ME, Ronksley PE, et al. Use of simulation-based education to improve outcomes of central venous catheterization: a systematic review and meta-analysis. *Acad Med*. 2011 Sep;86(9):1137-1147. PMID: 21785310

26. McKinney J, Cook DA, Wood D, Hatala R. Simulation-based training for cardiac auscultation skills: systematic review and meta-analysis. *J Gen Intern Med*. 2013 Feb;28(2):283-291. PMID: 22968795

27. McNaughton N, Ravitz P, Wadell A, Hodges BD. Psychiatric education and simulation: a review of the literature. *Can J Psychiatry*. 2008 Feb;53(2):85-93. PMID: 18357926

28. Mundell WC, Kennedy CC, Szostek JH, Cook DA. Simulation technology for resuscitation training: a systematic review and meta-analysis. *Resuscitation*. 2013 Sep;84(9):1174-1183. PMID: 23624247

29. Nestel D, Groom J, Eikeland-Husebo S, O'Donnell JM. Simulation for learning and teaching procedural skills: the state of the science. *Simul Healthc*. 2011 Aug;6 Suppl:S10-13. PMID: 21817857

30. O'Leary JA, Nash R, Lewis PA. High fidelity patient simulation as an educational tool in paediatric intensive care: A systematic review. *Nurse Educ Today*. 2015 Oct;35(10):e8-12. PMID: 26260524

31. Olejniczak EA, Schmidt NA, Brown JM. Simulation as an orientation strategy for new nurse graduates: an integrative review of the evidence. *Simulation in healthcare : journal of the Society for Simulation in Healthcare*. 2010;5(1):52-57.

32. Pulsford D, Jackson G, O'Brien T, Yates S, Duxbury J. Classroom-based and distance learning education and training courses in end-of-life care for health and social care staff: a systematic review. *Palliative medicine*. 2013;27(3):221-235.

33. Rakshasbhuvankar AA, Patole SK. Simulation-based training for neonatal resuscitation: A systematic review. *Journal of Paediatrics and Child Health*. 2014;50:27-28.

34. Rosen MA, Hunt EA, Pronovost PJ, Federowicz MA, Weaver SJ. In situ simulation in continuing education for the health care professions: a systematic review. *J Contin Educ Health Prof*. 2012 Fall;32(4):243-254. PMID: 23280527

35. Shearer JE. High-fidelity simulation and safety: an integrative review. *J Nurs Educ*. 2013 Jan;52(1):39-45. PMID: 23181458

36. Yuan HB, Williams BA, Fang JB, Ye QH. A systematic review of selected evidence on improving knowledge and skills through high-fidelity simulation. *Nurse Educ Today*. 2012 Apr;32(3):294-298. PMID: 21862186

37. Ali AA, Miller ET. Effectiveness of Video-Assisted Debriefing in Health Education: An Integrative Review. J Nurs Educ 2018 Jan 30;57(1):14–20. [doi: 10.3928/01484834-20180102-04]

38. AlReshidi N, Long T, Darvill A. A Systematic Review of the Impact of Educational Programs on Factors That Affect Nurses' Post-Operative Pain Management for Children. Comprehensive Child and Adolescent Nursing 2018 Jan 2;41(1):9–24. [doi: 10.1080/24694193.2017.1319432]

39. Ashraf H, Sodergren MH, Merali N, Mylonas G, Singh H, Darzi A. Eye-tracking technology in medical education: A systematic review. Medical Teacher 2018 Jan 2;40(1):62–69. PMID:29172823

40. Bakon S, Craft J, Wirihana L, Christensen M, Barr J, Tsai L. An integrative review of graduate transition programmes: Developmental considerations for nursing management. Nurse Education in Practice 2018 Jan;28:80–85. [doi: 10.1016/j.nepr.2017.10.009]

41. Fowler AC, Twigg D, Jacob E, Nattabi B. An integrative review of rural and remote nursing graduate programmes and experiences of nursing graduates. Journal of Clinical Nursing 2018 Mar 1;27(5–6):e753–e766. [doi: 10.1111/jocn.14211]

42. Gdanetz LM, Hamer MK, Thomas E, Tarasenko LM, Horton-Deutsch S, Jones J. Technology, Educator Intention, and Relationships in Virtual Learning Spaces: A Qualitative Metasynthesis. Journal of Nursing Education 2018 Apr 1;57(4):197–202. [doi: 10.3928/01484834-20180322-02]

43. Jansson M, Kääriäinen M, Kyngäs H. Effectiveness of Simulation-Based Education in Critical Care Nurses' Continuing Education: A Systematic Review. Clinical Simulation in Nursing 2013 Sep 1;9(9):e355–e360. [doi: 10.1016/j.ecns.2012.07.003]

44. Kaye SP. Nurses' Attitudes Toward Meaningful Use Technologies: An Integrative Review. CIN: Computers, Informatics, Nursing 2017 May;35(5):237. [doi: 10.1097/CIN.0000000000000310]

45. Martin P, Lizarondo L, Kumar S. A systematic review of the factors that influence the quality and effectiveness of telesupervision for health professionals. J Telemed Telecare 2018 May 1;24(4):271–281. [doi: 10.1177/1357633X17698868]

46. O'Rourke J, Horsley TL, Doolen J, Mariani B, Pariseault C. Integrative Review of Interprofessional Simulation in Nursing Practice. The Journal of Continuing Education in Nursing 2018 Feb 1;49(2):91–96. [doi: 10.3928/00220124-20180116-09]

47. Raman J. Mobile technology in nursing education: where do we go from here? A review of the literature. Nurse Education Today 2015 May;35(5):663–672. [doi: 10.1016/j.nedt.2015.01.018]

48. Rutherford-Hemming T, Lioce L. State of Interprofessional Education in Nursing: A Systematic Review. Nurse educator 2018 Jan;43(1):9–13. [doi: 10.1097/nne.0000000000000405]

Nursing outcomes not separable from other population (n=13)

1. Abelsson A, Rystedt I, Suserud BO, Lindwall L. Mapping the use of simulation in prehospital care - a literature review. *Scand J Trauma Resusc Emerg Med*. 2014 Mar 28;22:22. PMID: 24678868

2. Bluestone J, Johnson P, Fullerton J, et al. Effective in-service training design and delivery: evidence from an integrative literature review. *Hum Resour Health*. 2013 Oct 01;11:51. PMID: 24083659

3. Boonyasai RT, Windish DM, Chakraborti C, et al. Effectiveness of teaching quality improvement to clinicians: a systematic review. *Jama*. 2007 Sep 05;298(9):1023-1037. PMID : 17785647

4. Brydges R, Manzone J, Shanks D, et al. Self-regulated learning in simulation-based training: a systematic review and meta-analysis. *Med Educ*. 2015 Apr;49(4):368-378. PMID: 25800297

5. Fung L, Boet S, Qosa H, et al. The impact of simulation-based crisis resource management training: A systematic review. *Canadian Journal of Anesthesia*. 2013;60(1):S121.

6. Gjeraa K, Moller TP, Ostergaard D. Efficacy of simulation-based trauma team training of non-technical skills. A systematic review. *Acta anaesthesiologica Scandinavica*. 2014 Aug;58(7):775-787. PMID: 24828210

7. Liu Q, Peng W, Zhang F, et al. The Effectiveness of Blended Learning in Health Professions: Systematic Review and Meta-Analysis. *Journal of Medical Internet Research*. 2016;18(1):1-1. PMID: 112306705.

8. Schichtel M, Rose PW, Sellers C. Educational interventions for primary healthcare professionals to promote the early diagnosis of cancer: a systematic review. *Educ Prim Care*. 2013 Jul;24(4):274-290. PMID: 23906171

9. von Muhlen M, Ohno-Machado L. Reviewing social media use by clinicians. *Journal of the American Medical Informatics Association : JAMIA*. 2012 Sep-Oct;19(5):777-781. PMID: 22759618

10. Brown CL, Reygate K, Slee A, Coleman JJ, Pontefract SK, Bates DW, Husband AK, Watson N, Slight SP. A literature review of the training offered to qualified prescribers to use electronic prescribing systems: why is it so important? International Journal of Pharmacy Practice 2017 Jun 1;25(3):195–202. [doi: 10.1111/ijpp.12296]

11. Edirippulige S, Armfield N. Education and training to support the use of clinical telehealth: A review of the literature. J Telemed Telecare 2017 Feb 1;23(2):273–282. [doi: 10.1177/1357633X16632968]

12. Richmond H, Copsey B, Hall AM, Davies D, Lamb SE. A systematic review and meta-analysis of online versus alternative methods for training licensed health care professionals to deliver clinical interventions. BMC Medical Education [Internet] 2017 Dec [cited 2018 Oct 30];17(1). [doi: 10.1186/s12909-017-1047-4]

13. Vaona A, Banzi R, Kwag KH, Rigon G, Cereda D, Pecoraro V, Tramacere I, Moja L. E-learning for health professionals. The Cochrane database of systematic reviews 2018 Jan 21;1:Cd011736. [doi: 10.1002/14651858.CD011736.pub2]

No relevant outcomes on nursing care (n=11)

1. Booth A, Carroll C, Papaioannou D, Sutton A, Wong R. Applying findings from a systematic review of workplace-based e-learning: implications for health information professionals. *Health Info Libr J*. 2009 Mar;26(1):4-21. PMID: 19245639

2. Brydges R, Manzone J, Shanks D, et al. Self-regulated learning in simulation-based training: a systematic review and meta-analysis. *Med Educ*. 2015 Apr;49(4):368-378. PMID: 25800297

3. Cant RP, Cooper SJ. Simulation in the Internet age: the place of web-based simulation in nursing education. An integrative review. *Nurse Educ Today*. 2014 Dec;34(12):1435-1442. PMID: 25156144

4. Cheng A, Eppich W, Grant V, et al. Debriefing for technology-enhanced simulation: a systematic review and meta-analysis. *Medical education*. 2014;48(7):657-666.

5. Cook DA, Triola MM. Virtual patients: a critical literature review and proposed next steps. *Med Educ*. 2009 Apr;43(4):303-311. PMID: 19335571

6. Kleinpell R, Ely EW, Williams G, et al. Web-based resources for critical care education. *Critical Care Medicine*. 2011;39(3):541-553. PMID: 104823997

7. Pulsford D, Jackson G, O'Brien T, Yates S, Duxbury J. Classroom-based and distance learning education and training courses in end-of-life care for health and social care staff: a systematic review. *Palliative medicine*. 2013;27(3):221-235.

8. Roberts MJ, Perera M, Lawrentschuk N, et al. Globalization of continuing professional development by journal clubs via microblogging: a systematic review. *J Med Internet Res*. 2015 Apr 23;17(4):e103. PMID: 25908092

9. Wynne J. Nurse practitioner continuing education: exploring influences. *J Am Assoc Nurse Pract*. 2015 Jul;27(7):398-402. PMID: 25546117

10. Younger P. Internet-based information-seeking behaviour amongst doctors and nurses: a short review of the literature. *Health Info Libr J*. 2010 Mar;27(1):2-10. PMID: 20402799

11. Taroco ALC, Valente TC de O, Carbogim CS. Distance learning for updating health professionals in palliative care: a systematic review. BMJ Supportive & Palliative Care 2017 Jun 1;7(2):205–211. PMID:28062410

Context (n=4)

1. Elliott L, Decristofaro C, Carpenter A. Blending technology in teaching advanced health assessment in a family nurse practitioner program: Using personal digital assistants in a simulation laboratory. *Journal of the American Academy of Nurse Practitioners*. 2012;24(9):536-543.

2. Garrity MK, Jones K, VanderZwan KJ, de la Rocha AB, Epstein I. Integrative review of blogging: implications for nursing education. *J Nurs Educ*. 2014 Jul 01;53(7):395-401. PMID: 24972401

3. Raman J. Mobile technology in nursing education: where do we go from here? A review of the literature. Nurse Education Today 2015 May;35(5):663–672. [doi: 10.1016/j.nedt.2015.01.018]

4. Reisoğlu I, Topu B, Yılmaz R, Yılmaz TK, Göktaş Y. 3D virtual learning environments in education: a meta-review. Asia Pacific Educ Rev 2017 Mar 1;18(1):81–100. [doi: 10.1007/s12564-016-9467-0]

Langage (n=3)

1. Camacho AC. Analysis of national publications about distance education in nursing. *Revista brasileira de enfermagem*. 2009;62(4):588-593.

2. Crossetti MG, Bittencourt GK, Schaurich D, Tanccini T, Antunes M. Strategies for teaching the critical thinking abilities in nursing. *Revista gaúcha de enfermagem* 2009;30(4):732-741.

3. Veneroni L, Ferrari A, Carraro M, Clerici CA. [Online videos in the health field. Novel technologies for physicians and patients]. *Recenti Prog Med*. 2012 May;103(5):177-182. 22677943

No access to full text (n=2)

1. Benzel-Lindley J, Markantes T, Byron D, et al. Review of studies on simulation learning in occupational settings. Communicating Nursing Research. 2010 2010 Spring;43:589-589. PMID: 105080583.

2. Seifert AM, O'Neill M. Global Health Promotion. 2013;20(3):58-65.
