# Supplementary material for: Effects of E-Learning in a Continuing Education Context on Nursing Care: Systematic Review of Systematic Qualitative, Quantitative, and Mixed-Studies Reviews
Source: J Med Internet Res. 2019 Oct 2;21(10):e15118. doi: 10.2196/15118 (PMC6777280; doi:10.2196/15118)
Supplement: Multimedia Appendix 4 [file jmir_v21i10e15118_app4.pdf]

Multimedia appendix 4. General characteristics of included systematic reviews.

First author, year, location of first author	Purpose of the SR[a]	Type of SRs as reported by the SR authors	Type of SRs as classified by Rouleau et al.	Numbers of primary studies about nurses using e-learning interventions in a CE[b] context / Total of primary studies in the SR	Search date	Number of nurses and their roles (when specified and reported) and their settings
Bloomfield (2008), United Kingdom [36]	To review the research investigating computer-assisted learning for clinical skills education in nursing, the ways in which it has been studied and the general findings.	Integrative review	QT[c]	3/12	1997 to 2006	171 nurses<br><br>Settings: General hospital and surgical units
Brunero (2012), Australia [37]	To review and synthesize research evidence on Mental Health Education Programmes that have been designed to develop the knowledge, skills and attitudes of general healthcare professionals.	Integrative review	MSR[d]	2/25	1990-01 to 2010-12	273 nurses (including mental health liaison nurses)<br><br>Setting: NR[e]
Byrne (2018), United Kingdom [38]	To compare the methods used to train staff in clinical skills.	SR	QT	1/10	Initial search: 2006 Repeated checks up to June 2007	28 nurses<br><br>Setting: NR
Carroll (2009) United Kingdom [39]	To address the following question: Which e-learning techniques most enhance the	SR of qualitative data	Unclear but probably MSR considering data collection	4/19	1992 to the end of 2007	93 nurses<br><br>Settings: University, college

	learning experience of health professionals in the United Kingdom?		methods (e.g. surveys and interviews)			
Chipps (2012), South Africa [40]	1) To systematically review the literature and critique the research methods on videoconference-based education for the education of doctors and nurses<br>2) To summarize the existing evidence on the effectiveness of videoconference education for medical and nursing staff.<br>3) To apply the findings to South Africa.	SR	QT	2/5	1990 to 2011	20 palliative care nurses and 12 clinical nurse specialists<br><br>Setting: NR
Coyne (2018), Australia [41]	To inform future educational strategies by synthesising research related to blended learning resources using simulation videos to teach clinical skills for health students.	Integrative review	MSR	1/10	2006 to 2016	22 nurses<br><br>Setting: Stroke rehabilitation
Du (2013), China [42]	To examine the efficacy of web-based distance education for nursing students and employed nurses.	SR	QT	3/9	Up to 2012-07	193 nurses<br><br>Settings: Neurological unit, geriatric hospital, ED[g]
Feng (2013), Taiwan [43]	To determine the effectiveness of situated e-learning in prelicensure and postlicensure medical and	SR	QT	2/14	2001-01 to 2012-05	80 school nurses and nurses<br><br>Setting: ED

	nursing education.					
Freire (2015), Brazil [44]	To identify resources that support education strategies mediated by technology in neonatal nursing.	SR	MSR	2/9	Up to 2014-01	104 nurses<br><br>Setting: Neonatal units
Härkänen (2016), Finland [45]	To evaluate the nature, quality and effectiveness of educational interventions designed to increase the medication administration skills and safety of registered nurses working in hospitals.	SR with meta-analysis	QT	4/14	2000-01 to 2015-04	Nurses: NR<br><br>Settings: Medicine and surgical units, general hospital setting
Hegland (2017), Norway [46]	To evaluate effect of simulation-based training on nurses' skills and knowledge.	SR	QT	2/15	No restriction	171 nurses<br><br>Settings: Medical and surgical units, primary care
Hines (2015), Australia [47]	To identify the effectiveness of workplace, tertiary-level educational, or other interventions designed to improve or increase postregistration nurses' understanding of research literature and ability to critically interact with research literature with the aim of promoting the use of research evidence in practice in comparison to no intervention, other	SR	QT	1/10	No restriction	Nurses: NR<br><br>Settings: Workplace environments in variety of clinical specialties

	intervention, or usual practice.					
Kakushi (2016), Brazil [48]	To identify the use of social networking in nursing education.	Integrative literature review	Unclear - MSR (Authors didn't precise the type of evidence)	1/14	Up to 2015-04	Setting: Neonatal unit
Kang (2017), Republic of Korea [49]	To evaluate the effects of web-based nursing education programs by analyzing articles that report on how such programs affect learners' knowledge and clinical performance levels.	SR with meta-analyses	QT	5/11	2000-01 to 2016-07	454 nurses<br><br>Setting: NR
Knapp (2008), USA [10]	To review the literature systematically regarding the use of the Internet in nursing staff development and to focus specifically on the potential usefulness of this method in helping critical nurses understand how they can help patients' families during extremely stressful situations.	SR	MSR	5/5	Unspecified (articles range from 2001-2006)	310 nurses (when specified)<br><br>Settings: Neurosurgical unit, surgical intensive care unit, general hospital settings
Lahti (2014), Finland [1]	To investigate the impact of e-learning on knowledge, skills and satisfaction among nurses and nursing students compared to traditional	SR with meta-analysis	QT	2/11	1948 to 2010-12	130 nurses<br><br>Settings: Nursing management rooms with computers hospital training room, hospital and

	education methods.					nursing facilities
Lam-Antoniades (2009), Canada [50]	To provide an update on evidence from RCTs[h] assessing the effectiveness of electronic continuing education in the health professions.	Literature search of RCTs	QT	1/15	2004-01 to 2007-12	81 nurses<br><br>Setting: NR
Lawn (2017), Australia [51]	To examine what is known about the evidence of e-learning instructional designs and formats that are best for teaching the depth of self-management skills needed by health professionals to work with patients with chronic and complex care need.	Integrative review	MSR	2/10	2006-2016	500 practice nurses serving veterans; nurses (NR) serving hospital inpatients<br><br>Settings: Primary care, medical surgical units, telemetry unit
Nicoll (2018), United Kingdom [3]	To identify the current literature relating to the evaluation of technology-enhanced learning programs for health care professionals and to critically appraise the quality of the studies.	Systematic integrative review	MSR	4/21	2006-01 to 2017-01	658 nurses, oncology nurses, practice nurses
Phillips (2012), Australia [52]	To review published studies evaluating the impact of continuing professional development programmes on rural nurses palliative care capabilities in	Integrative review	MSR	2/10	1993-01 to 2010-06	63 nurses<br><br>Settings: Rural/remote settings, community

	order to inform the development of targeted learning activities for this population.					
Sinclair (2016), Australia [4]	To identify, appraise and synthesise the best available evidence for the effectiveness of e-learning programmes on health care professional behavior and patient outcomes.	SR	QT	2/7	2004-01 to 2015-07	90 nurses<br><br>Settings: School, emergency department
Tomlinson (2013), Australia [53]	To determine whether tele-learning delivery methods achieve equivalent learning outcomes when compared with traditional face-to-face education delivery methods.	SR	QT	1/13	2000-01 to 2012-12	15 community nurses<br><br>Setting: Community

[a] SR: systematic reviews

[b] CE: continuing education

[c] QT: quantitative reviews

[d] MSR: mixed studies reviews

[e] NR: not reported

[f] QL: qualitative reviews

[g] ED: emergency departments

[h] RCTs: randomized controlled trials
