# Supplementary material for: Effects of E-Learning in a Continuing Education Context on Nursing Care: Systematic Review of Systematic Qualitative, Quantitative, and Mixed-Studies Reviews
Source: J Med Internet Res. 2019 Oct 2;21(10):e15118. doi: 10.2196/15118 (PMC6777280; doi:10.2196/15118)
Supplement: Multimedia Appendix 5 [file jmir_v21i10e15118_app5.pdf]

**Multimedia Appendix 5.** Interventions, comparisons and level of evaluation/outcomes.

| First author (year) | Examples of interventions | | Examples of comparisons | Levels of evaluation according to Kirkpatrick Model [30] and description of outcomes |
|---|---|---|---|---|
| | **Examples of nursing topics** | **Examples of medium used** | | |
| Bloomfield (2008) [36] | Universal precautions and responded patient situations; IV[a] injections on basic oral medication administra-tion. | CAI[b], CD-ROM, CAL[c] | Face-to-face learning: Lecture on hospital orientation<br><br>No intervention | **I:** 1/3 studies found high level of satisfaction with computer-assisted learning.<br><br>**II:** 3/3 studies found positive outcomes related to skills (universal precautions, IV injections, medication administration). 2/3 studies found no significant outcomes related to learning on the topics of IV injections as well as medication administration and preparation. |
| Brunero (2012) [37] | Demanding behavior, manipula-ting, splitting and attention seeking behavior; depression assessment, treatment, care planning, communica-tion, and referral methods. | e-learning based education program, case-based learning | No comparison | **II:** 2/2 studies were positive. 1 study found that skills and knowledge were improved, 1 study pointed out improvements in nurses confidence and in reducing stress in nurse-patient relationship, 1 study showed improvements in non-technical skills such as communication, activity scheduling and assessment, and 1 study saw improvements in clinical skills (i.e. monitoring and care practice).<br>**IV:** 1/2 studies had positive outcome related to care for older adults as perceived by nurses.<br>**Other:** 1/2 studies found that CD-ROM-based education reduced costs. |
| Byrne (2018) [38] | Venous cannulation | Computer-based simulation | Non-electronic intervention: plastic arm | **II:** 1/1 study found lower success with cannulation when using computer-based stimulation. |
| Carroll (2009) [39] | NR | e-learning courses | N/A | **I**: 4/8 studies found satisfaction with e-learning. Nurses had a good learning experience, they found confidence in the |

| | | | | |
|---|---|---|---|---|
| | | | | medium, felt a sense of belonging and embraced anonymity as well as scope of reflection. |
| Chipps (2012) [40] | Palliative care education and advanced health assessment | Videoconfe-rencing and workshops | Face-to-face learning | **I:** 1/2 study found dissatisfaction of using e-learning (preference for face-to-face, technical difficulties). 1/2 studies pointed out the importance of interactions during sessions.<br><br>**II:** 1/2 study found improvement in knowledge regarding assessment with e-learning. 1/2 found no significant change in nurses' knowledge related to pain, physical and psychological symptoms as well as loss. |
| Coyne (2018) [41] | Dysphagia training: modifying fluids, assisting with feeding and swallowing. | Blended learning using videos | No comparison | **I & II:** In 1/1 study, nurses found that the presentation of information in a blended learning format provided a platform for discussion and extended learning with peers and tutors. Nurses' learning effectiveness improved significantly: dysphagia knowledge and attitudes achieved significance. |
| Du (2013) [42] | NR | Web-based distance learning | Face-to-face learning (e.g. Instructor-led videotaped learning program, CPR[e]-defibrillation course) | **I:** 1/3 study found higher satisfaction with e-learning than videotape courses.<br><br>**II:** 1/3 study found improvement in knowledge regarding assessment of ability of neurological function with e-learning. 1/3 study had a higher self-efficacy and performance score in the e-learning intervention. 1/3 study found that CPR and defibrillation performance was worse with e-learning. |
| Feng (2013) [43] | Emergency preparedness and child | Situated e-learning | No intervention | **II:** 2/2 studies found positive effects: one study improved knowledge compared to no intervention, and the other found |

| | | | | |
|---|---|---|---|---|
| | abuse detection | | | that nurses had better performance outcomes with e-learning compared to no intervention. |
| Freire (2015) [44] | Semiotics and semiology of the preterm newborn; on-line neonatal training and orientation program | Distance education about virtual learning object | No comparison | **I:** In 2/2 studies, nurses evaluated positively e-learning, for example, regarding the quality of content.<br><br>**II:** 1/2 study had positive outcomes with an increase in skills score with e-learning and in knowledge related to neonatal care. |
| Härkänen (2016) [45] | Medication administration skills and safety | Online programs and interactive CD-ROM program on | Face-to-face learning<br>No intervention<br>No comparison | **II:** 1/4 study revealed less error rates for nurses who participated in CD-ROM program regarding medication administration and preparation. 3/4 studies had mixed outcomes regarding knowledge on medication administration and calculation: 2/4 found positive outcomes and 1/4 study found that classroom was superior to e-learning for drug dose calculations. |
| Hegland (2017) [46] | Medication administra-tion | Interactive CD-ROM<br>e-Learning modules | No additional training<br>Face-to-face learning and 1-day self-study | **II:** 2/2 studies had mixed outcomes on medication administration and preparation: one study was in favor of e-learning intervention and the other one had no effect. The meta-analysis on computer-based simulation compared to other learning strategies showed significant effect in favour of e-learning while non-significant difference in knowledge scores [drug dose calculation] has been found between groups. |
| Hines (2015) [47] | Research literacy, critical appraisal competencies | Virtual journal club in "Second Life" virtual environment (Online program) | No comparison (pre/post) | **II:** 1/1 study revealed that a virtual learning environment, improved nurses' competencies in appraising many components of research literature (e.g. determining designs), but not in the one of identifying samples. |

| | | | | |
|---|---|---|---|---|
| Kakushi (2016) [48] | NR | Face-to-face and online learning with social networking (Ning), by means of audio, videos, quizzes, animations and forums. | No comparison: none specified | **I:** In 1/1 study, newly hired nurses from a neonatal hospital evaluated their experience with e-learning intervention positively through social interaction and active learning. |
| Kang (2017) [49] | NR | Web-based learning | Traditional lecture | **II:** When the participants were nurses, 5/11 studies reported a positive effect on knowledge; however, the effect size difference was not significant. |
| Knapp (2008) [10] | Stress management interventions among families in critical care settings | Intranet learning; online program; computer with Internet access | Non-electronic intervention<br>No comparison | **I:** In 2/5 studies, nurses were satisfied with e-learning interventions: they appreciated the flexibility, effectiveness and convenience of the technology and they were pleased with the quality of support received. In 2/5 studies, nurses were dissatisfied with e-learning, explained by a lack of computer experience and Internet literacy, computer issues and by slower information exchange.<br><br>**II:** In 2/5 studies, nurses improved knowledge of the Physiology and Chronic health evaluation and they broadened knowledge through Internet access. In 1/5 study, there was no significant change in improving knowledge.<br><br>**Other:** In 1/5 study, Intranets were a low-cost method of providing education for their nursing staff. |
| Lahti (2014) [1] | Hospital quality, IV injections | Computer programme, e-learning | Traditional learning | **Note:** Only "usable data" as reported by authors were extracted.<br>**II:** In 2/2 studies, nurses gained knowledge regarding IV injections and hospital quality. |

| | | | | |
|---|---|---|---|---|
| Lam-Antoniades (2009) [50] | IV insertion | CD-ROM multicomponent (text, graph, picture, film, sound) | Face-to-face learning (lecture) | **I:** In 1/1 study, nurses found advantages of e-continuing education over lecture courses. |
| Lawn (2017) [51] | Use of brief motivational interviewing as a communication style to influence health behavior change | Online learning module regarding interactive modules offered online | (Qualitative evaluation and pre/post-test assessment) | **I**: In 2/2 studies, nurses were satisfied with e-learning interventions and valued it for the following reasons: patient-centred approach, flexibility, time saving, and self-directed learning. They stressed the importance of authentic scenarios and of practicing skills in the work context. In 1/2 study, nurses identified these challenges: access, navigation, and time.<br>**II:** In 1/2 study, nurses had positive attitudes toward and statistically significant increase in mean score about effectiveness of online learning modules for motivational interviewing. |
| Nicoll (2018) [3] | Arterial blood gas interpretation<br><br>Motivational interviewing | Technology enhanced learning (e.g. Web-based e-learning, learning management systems). | No comparison<br>No additional training | **I:** In 2/4 studies, nurses felt satisfied with ELP[f] and TICP[g]. They found the e-learning platform for brief motivational interviewing was feasible and acceptable in cardiovascular care.<br>**II:** 4/4 studies had positive outcomes on knowledge and clinical skills. Staff nurses increased knowledge of arterial blood gas interpretation. They found e-learning intervention focusing on a rare disease in a culturally distinct population as an effective educating strategy. Nurses perceived skill and clinical use of brief motivational interviewing more favourable (post-training). |
| Phillips (2012) [52] | Palliative care delivery | Videoconferencing<br><br>Email | Face-to-face learning<br>No comparison (pre/post) | **I:** In 1/2 study, nurses preferred face-to-face to videoconferencing format.<br><br>**II:** In 2/2 studies, nurses |

| | | | | |
|---|---|---|---|---|
| | | | | perceived benefits on their knowledge in palliative care and on personal and professional development when using e-learning, while no significant change was reported in one study. |
| Sinclair (2016) [4] | Emergency preparedness skills performance<br><br>Child abuse detection | e-Learning modules | No intervention | **II:** In 2/2 studies, nurses improved clinical skills when they used e-learning. They had better emergency preparedness with e-learning than no intervention and they improved child abuse detection with e-learning compared to no intervention. |
| Tomlinson (2013) [53] | NR | Videoconfe-rencing | Face-to-face learning | **II:** In 1/1 study, there was no significant increase in learning effectiveness with videoconferencing compared to face-to-face. |

[a] IV: intravenous

[b] NR: not reported

[c]: CAI: computer-assisted instruction

[d:] CAL: computer-assisted learning

[e] CPR: cardiopulmonary resuscitation-defibrillation

[f] ELP: e-learning programmes

[g] TICP: traditional in-classroom programme
